# Emerging therapeutic frontiers in cancer: insights into posttranslational modifications of PD-1/PD-L1 and regulatory pathways

**DOI:** 10.1186/s40164-024-00515-5

**Published:** 2024-04-23

**Authors:** Rong Wang, Shiwei He, Jun Long, Yian Wang, Xianjie Jiang, Mingfen Chen, Jie Wang

**Affiliations:** 1 Department of Pathology, Institute of Oncology, The School of Basic Medical Sciences & Diagnostic Pathology Center, Fujian Medical University, Fuzhou, Fujian China; 2 Shenzhen Geim Graphene Center, Tsinghua-Berkeley Shenzhen Institute & Tsinghua Shenzhen International Graduate School, Tsinghua University, Shenzhen, Guangdong China; 3 School of Basic Medical Sciences, Hebei Medical University, Shijiazhuang, Hebei China; 4 The Key Laboratory of Model Animals and Stem Cell Biology in Hunan Province, School of Medicine, The Engineering Research Center of Reproduction and Translational Medicine of Hunan Province, Hunan Normal University, Changsha, Hunan China; 5 Hunan Key Laboratory of Cancer Metabolism, Hunan Cancer Hospital and the Affiliated Cancer Hospital of Xiangya School of Medicine, Central South University, Changsha, Hunan China; 6 Department of Radiation Oncology, The Second Affiliated Hospital of Fujian Medical University, Fujian Medical University, Quanzhou, Fujian China

**Keywords:** PD-1, PD-L1, Posttranslational modification, Immunotherapy

## Abstract

The interaction between programmed cell death ligand 1 (PD-L1), which is expressed on the surface of tumor cells, and programmed cell death 1 (PD-1), which is expressed on T cells, impedes the effective activation of tumor antigen-specific T cells, resulting in the evasion of tumor cells from immune-mediated killing. Blocking the PD-1/PD-L1 signaling pathway has been shown to be effective in preventing tumor immune evasion. PD-1/PD-L1 blocking antibodies have garnered significant attention in recent years within the field of tumor treatments, given the aforementioned mechanism. Furthermore, clinical research has substantiated the efficacy and safety of this immunotherapy across various tumors, offering renewed optimism for patients. However, challenges persist in anti-PD-1/PD-L1 therapies, marked by limited indications and the emergence of drug resistance. Consequently, identifying additional regulatory pathways and molecules associated with PD-1/PD-L1 and implementing judicious combined treatments are imperative for addressing the intricacies of tumor immune mechanisms. This review briefly outlines the structure of the PD-1/PD-L1 molecule, emphasizing the posttranslational modification regulatory mechanisms and related targets. Additionally, a comprehensive overview on the clinical research landscape concerning PD-1/PD-L1 post-translational modifications combined with PD-1/PD-L1 blocking antibodies to enhance outcomes for a broader spectrum of patients is presented based on foundational research.

## Introduction

Programmed cell death 1 (PD-1) and its ligand PD-L1 have become pivotal in advancing tumor treatment by effectively modulating immune responses [1]. PD-L1 is expressed across various tumors, while PD-1 is primarily expressed on T cells within tumor tissues [2]. PD-L1 engages with PD-1, creating a molecular barrier that inhibits the cytotoxic actions of immune cells [3]. Overcoming this inhibition is possible through blocking antibodies or recombinant proteins that target signaling pathways, reactivating immune responses. Monoclonal antibodies against PD-1 and PD-L1 have demonstrated significant therapeutic success, indicating that immune checkpoint blockade therapy is a potent antitumor treatment. However, its current use mainly as a second-line treatment for advanced tumors and the emergence of drug resistance highlight ongoing challenges [4]. These factors underscore the necessity for continued research to potentially expand its use earlier in treatment protocols.

Exploring new biomarkers and developing combination drug therapies are essential for combating these challenges. Research has shown that PD-1 transcription can be increased by activating B-cell CLL/lymphoma 6 (BCL6), and various elements, such as cytokines, hypoxia, bromodomain-containing protein 4 (BRD4), and noncoding RNA, can elevate PD-L1 expression by influencing transcription [5, 6]. With advancements in detection technologies, numerous posttranslational modifications (PTMs) have been identified that play critical roles in human diseases and offer avenues for new treatments. Recent studies have focused on PTMs that impact PD-1/PD-L1 protein expression and their roles in immunosuppression. PD-1/PD-L1 is negatively regulated by mechanisms such as phosphorylation, ubiquitination, ubiquitin-like modification and methylation. Conversely, positive regulation occurs through processes such as deubiquitination, glycosylation, palmitoylation, adenosine diphosphate (ADP) ribosylation, and deacetylation [7]. A deeper understanding of these regulatory mechanisms and identification of novel targets for PD-1/PD-L1 modification are vital for advancing tumor immunotherapy toward precise treatments. Moreover, ongoing efforts are needed to discover and test safe, effective drug combinations to improve therapeutic outcomes.

## Structures of PD-1/PD-L1 and their potential sites modulate PTMs

**Fig. 1:**
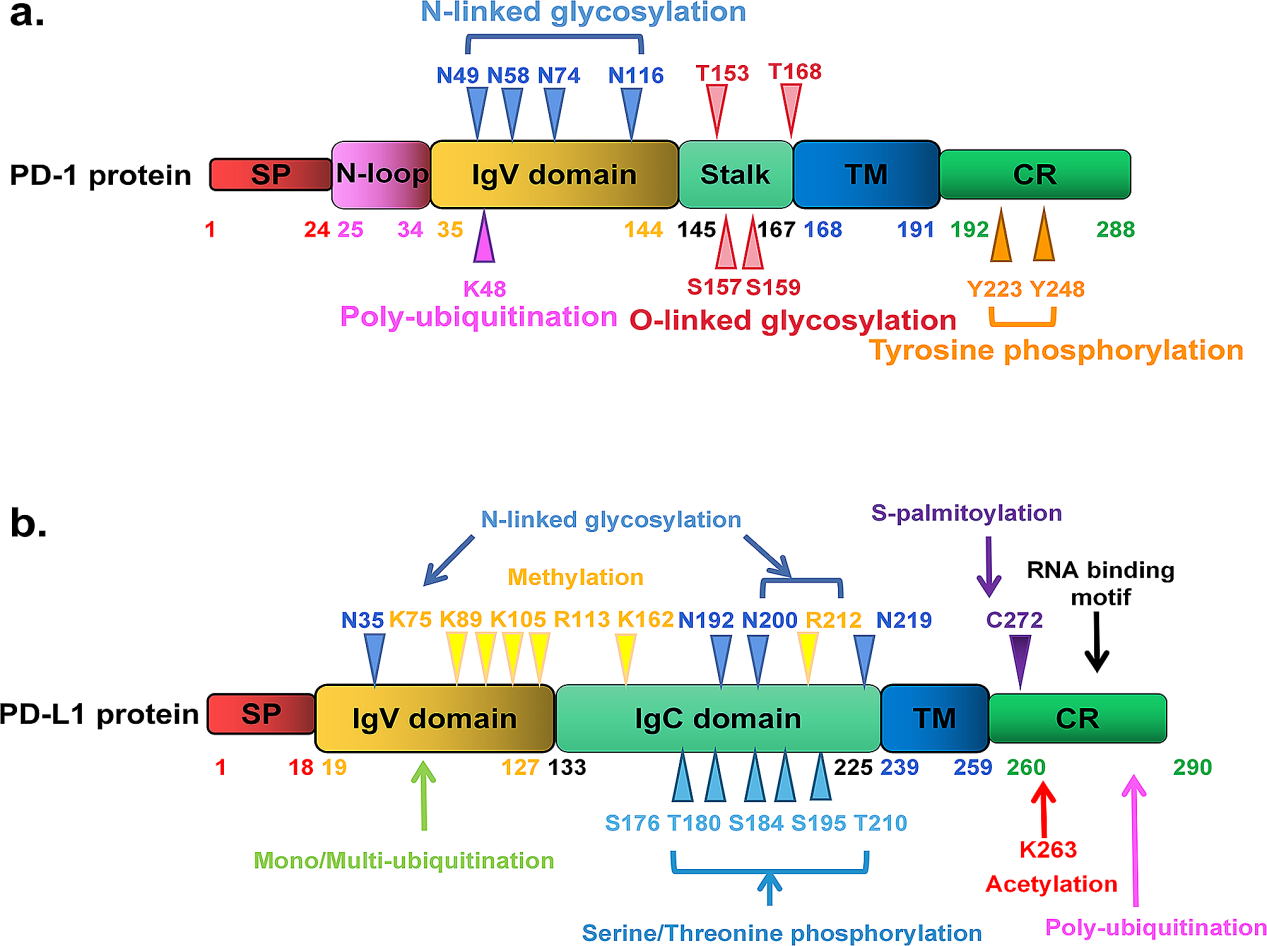
Schematic of PD-1/PD-L1 proteins highlighting potential PTM sites on PD-1/PD-L1. **a** The full-length PD-1 protein is divided into three segments: the ectodomain, transmembrane (TM), and a cytoplasmic region (CR). The ectodomain comprises the signal peptide (SP), N-loop, IgV domain, and stalk region, with amino acid positions denoted by numbers. Potential N-glycosylation sites at N49, N58, N74, and N116 are marked with blue arrowheads. Yellow arrowheads indicate potential tyrosine phosphorylation sites at Y223 and Y248. The poly-ubiquitination site at K48 is denoted by pink arrowheads. O-glycosylation sites at T153, S157, S159, and T168 are indicated with red arrows. **b** Full-length PD-L1 is divided into an ectodomain, transmembrane, and cytoplasmic region. The ectodomain comprises the signal peptide, IgV, and IgC domains, with amino acid positions indicated by numbers. Potential N-glycosylation sites at N35, N192, N200, and N219 are marked with blue arrowheads. Serine/threonine phosphorylation sites, located at S176, T180, S184, S195, and T210, are denoted by orange arrowheads. The S-palmitoylation site at C272 is indicated with a purple arrowhead. The mono/multiubiquitination motif is situated in the IgV domain, while the polyubiquitination site is in the cytoplasmic region of PD-L1. The acetylation site K263 is highlighted with a red arrow. Methylation sites at K75, K89, K105, R113, K162, and R212 are indicated with yellow arrowheads

PD-1 (CD279), encoded by the *PDCD1* gene on chromosome 2q37.3, is a type I transmembrane protein from the immunoglobulin superfamily, specifically the CD28 family. Unlike its family members, PD-1 uniquely exists as a monomer expressed on activated T cells, B cells, NK T cells, monocytes, and some dendritic cells (DCs) [8, 9]. It consists of 288 amino acids and features an ectodomain with a signal peptide, an N-loop, an IgV-like domain (with four N-linked glycosylation sites: N49, N58, N74, and N116, and a polyubiquitination site at K48), a transmembrane domain, and a cytoplasmic region with the key signaling motifs ITIM and ITSM and phosphorylation sites at Y223 and Y248 [10–14]. Recent research has identified additional O-glycosylation sites (T153, S157, S159, and T168) in the stalk region, indicating complex posttranslational modifications [15]. PD-1 interacts with two ligands, PD-L1 and PD-L2, from the B7 family, with PD-L2 binding to PD-1 with over three times the affinity of PD-L1 [16]. PD-L2 is distributed primarily on activated DCs and some macrophages, whereas PD-L1, which is more widely expressed on both immune and tumor cells, plays a crucial role in tumor immunity [17]. PD-L1 is a 40-kDa glycoprotein encoded by the *CD274* gene on chromosome 9p24.1. Its structure includes a signal peptide, extracellular IgV and IgC domains, a transmembrane domain, and a cytoplasmic region. The extracellular regions contain four glycosylation sites (N35, N192, N200, and N219) [18], while the IgC domains have five phosphorylation sites (S176, T180, S184, S195, and T210) [19, 20]. The cytoplasmic tail includes an S-palmitoylation site at C272 [21], six methylation sites (K75, K89, K105, R113, K162, and R212) [22], and an acetylation site at K263 [23]. Moreover, recent studies have shown that the PD-L1 intracellular domain functions as an RNA binding protein [24] (Fig. 1).

## Preclinical study of PTMs inhibiting PD-L1 expression and function

### PD-L1 phosphorylation inhibits PD-L1 protein expression by mediating ubiquitination

The process of protein phosphorylation involves the transfer of a phosphate group from ATP to amino acid residues of the target protein catalyzed by a series of protein kinases. This modification primarily occurs on two types of amino acids: serine (Ser or S) and threonine (Thr or T), as well as tyrosine (Tyr or Y). Protein phosphorylation plays a crucial role in regulating the activity of enzymes and other essential functional molecules, facilitating second messenger delivery and initiating enzymatic cascade reactions [25].

**Fig. 2:**
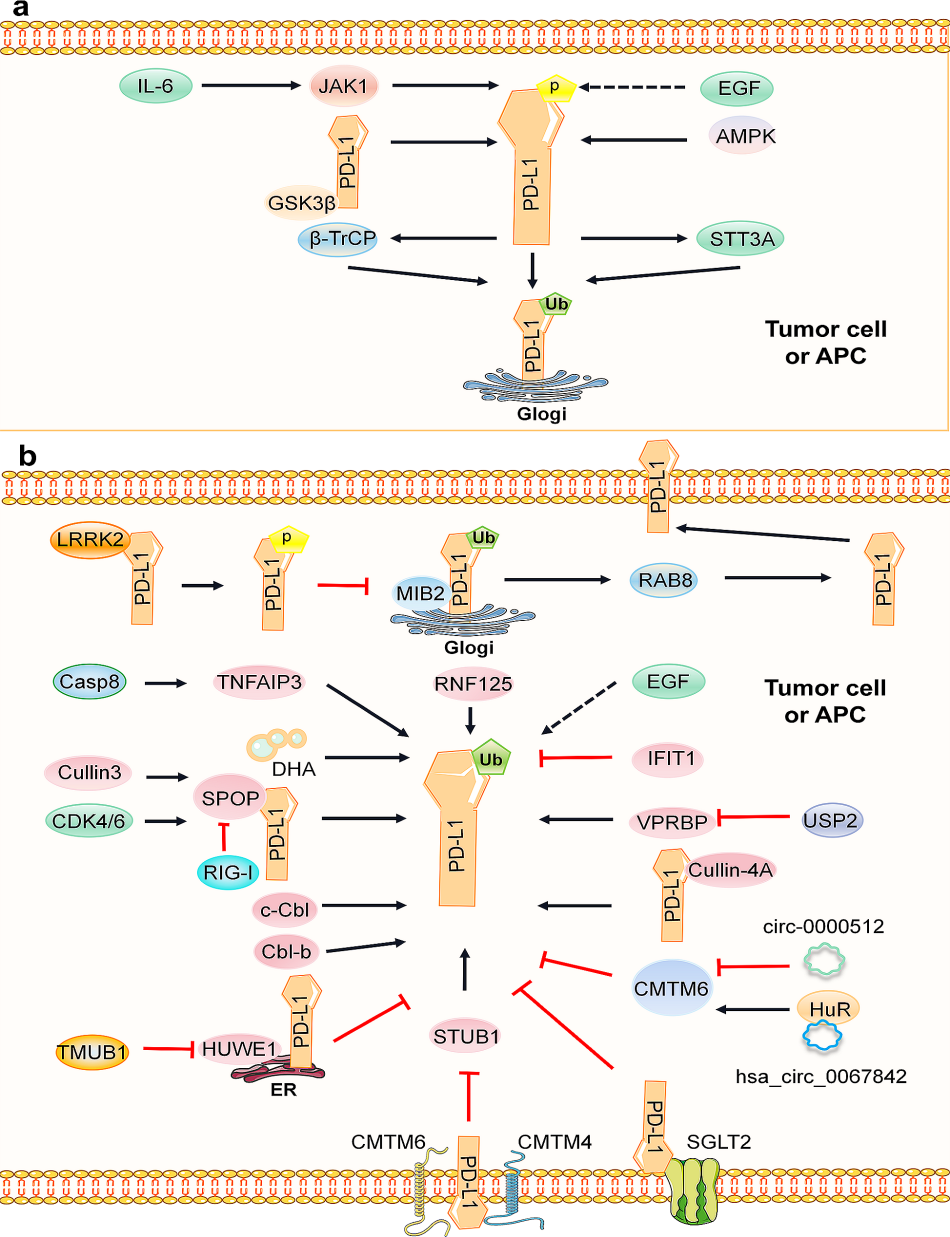
Regulatory pathways governing PD-L1 via phosphorylation and ubiquitination. **a** In combination with JAK1, GSK3β, AMPK, EGF, STT3A, and IL-6 collaboratively facilitate serine/threonine phosphorylation of PD-L1, leading to subsequent ubiquitination. **b** β-TrCP, C-Cbl, Cbl-b, and DHA contribute to the increase in PD-L1 ubiquitination. Conversely, IFIT1 impedes PD-L1 ubiquitination. CDK4/6 enhances PD-L1 ubiquitination via SPOP. The E3 ubiquitin ligase HUWE1, in conjunction with PD-L1, facilitates PD-L1 ubiquitination. STUB1 stimulates PD-L1 ubiquitination, leading to subsequent degradation, while CMTM6/4 impedes the binding of PD-L1 to STUB1, thereby downregulating PD-L1 ubiquitination. Casp8 promotes PD-L1 ubiquitination by upregulating TNFAIP3. USP2 modulates the stability of VPRBP and facilitates PD-L1 ubiquitination. MIB2 catalyzes PD-L1 ubiquitination, mediating the trafficking of PD-L1 from the trans-Golgi network to the membrane through RAB8. Circular RNA-0000512 promotes PD-L1 ubiquitination by suppressing CMTM6 expression, while circular RNA-0067842 inhibits PD-L1 ubiquitination by upregulating CMTM6. The black arrows indicate positive regulation, and the red arrows indicate negative regulation

The nonglycosylated PD-L1 protein exhibits extreme instability, with a half-life of approximately 4 h. Serine/threonine phosphorylation of nonglycosylated PD-L1 mediates its ubiquitination and subsequent degradation. The interleukin 6 (IL-6)/Janus kinase 1 (JAK1) pathway phosphorylates PD-L1 at Y112, facilitating its binding to the N-glycosyltransferase STT3A, thus preventing PD-L1 ubiquitination and degradation [26]. Glycogen synthase kinase 3β (GSK3β), a serine/threonine protein kinase, initiates the phosphorylation of PD-L1 at sites S176, T180, and S184. This phosphorylation triggers the interaction with the E3 ligase β-TrCP, which targets proteins for proteasome degradation [27]. Additionally, D-mannose activates AMP-activated protein kinase (AMPK), leading to further phosphorylation of PD-L1 at S195 and proteasome degradation [28]. (Fig. 2).

### The expression of PD-L1 is negatively regulated by ubiquitination

Ubiquitination is the process of covalently attaching ubiquitin to a target protein under the catalysis of a series of enzymes. In monoubiquitination, a target protein binds to a single ubiquitin molecule. Multiubiquitination is the process by which a single ubiquitin molecule labels multiple lysine residues of a target protein. Polyubiquitination, on the other hand, occurs when multiple ubiquitin molecules label a single lysine residue of the target protein [29–31]. The E3 ligase plays a crucial and specific role in this process by regulating the activity of the ubiquitination system in these enzymatic cascades [29].

The degradation of PD-L1 is intricately regulated by various ubiquitination-dependent proteasome pathways. Previous research has demonstrated that EGF can promote the expression of PD-L1 [32]. Horita et al. revealed that epidermal growth factor (EGF) induced PD-L1 monoubiquitination and polyubiquitination prior to EGF-mediated PD-L1 protein expression. Treatment of skin squamous carcinoma cells with gefitinib and SCH772984, chemical inhibitors of the EGF receptor (EGFR) pathway, inhibited the monoubiquitination and polyubiquitination of PD-L1 [33]. The chemical inhibitor PYR41 was also found to prevent the EGF-mediated increase in PD-L1 protein levels by inhibiting E1 ubiquitinase [33]. The E3 ubiquitinases Cbl-b and c-Cbl were shown to be involved in the downregulation of PD-L1 in EGFR wild-type non-small cell lung cancer (NSCLC) [34]. Cyclin-dependent kinase 4 (CDK4) and Cullin 3- speckle-type POZ protein (SPOP), an E3 ligase bound to Cullin3, can regulate the protein level of PD-L1 through the classical proteasome-mediated degradation pathway [35]. As mentioned in the PD-L1 phosphorylation section above, resveratrol can promote the GSK3β-β-TrCP-mediated degradation of polyubiquitinated PD-L1 [27]. Sun et al. proposed that leucine-rich repeat kinase 2 (LRRK2) inhibits ubiquitin‒proteasome degradation through the phosphorylation of PD-L1 [20]. Gao et al. reported that *Fusobacterium nucleatum*, which is enriched in colorectal cancer tissues, upregulates the expression of PD-L1 by reducing the ubiquitination-mediated degradation of PD-L1 through IFIT1 [36]. RIG-I can compete with SPOP for binding to PD-L1, leading to reduced polyubiquitination and proteasome degradation of PD-L1 [37]. Ring finger protein 125 (RNF125) promotes ubiquitin-mediated degradation of PD-L1 and downregulates PD-L1 in oral squamous cell carcinoma (OSCC) TSCCA cells [38]. Transmembrane and ubiquitin-like domain-containing protein 1 (TMUB1) inhibits PD-L1 K281 polyubiquitination in the endoplasmic reticulum (ER) by interacting with PD-L1 and competing with HECT, UBA, and WWE domain protein 1 (HUWE1), an E3 ubiquitin ligase [39]. Researchers have designed and synthesized PTPR, a peptide that competes with PD-L1 and weakens the regulatory role of TMUB1 at the cellular level [39]. As a substrate recognition subunit of the Cullin-4 (CUL4)-damage-specific DNA binding protein 1 (DDB1) ubiquitin E3 ligase complex, VPRBP directly induces ubiquitin-mediated PD-L1 degradation, and the stability of VPRBP is controlled by USP2 [40]. Docosahexaenoic acid (DHA) promotes PD-L1 degradation through the ubiquitin‒proteasome pathway, leading to decreased PD-L1 expression. This, in turn, reduces the PD-L1 and PD-1 interaction, reversing PD-L1-mediated immunosuppression and further promoting tumor growth inhibition [41]. Furthermore, the interaction between PD-L1 and Cullin-4 A facilitates the ubiquitination of PD-L1 [42]. Casp8 induces PD-L1 ubiquitination and promotes its degradation by upregulating TNFAIP3 (A20) expression in murine melanoma, suggesting that reduced Casp8 expression may serve as a potential biomarker for predicting sensitivity to anti-PD-L1 immunotherapies [43, 44]. (Fig. 2)

The ubiquitination of PD-L1 is not confined to the ER and Golgi apparatus but also involves the promotion of lysosomal degradation by endosomes. Mezzadra et al. demonstrated that CKLF-like MARVEL transmembrane domain-containing protein (CMTM)6 and CMTM4 bind to the ectodomain of PD-L1, preventing lysine residues in the cytoplasmic tail from interacting with the E3 ubiquitin ligase STUB1 [45]. This interference disrupts polyubiquitination, extending the half-life of the PD-L1 protein. Subsequently, CMTM6 enhances PD-L1 expression and inhibits the killing effect of tumor-specific T cells in mouse melanoma models [45]. Additionally, Burr et al. reported that CMTM6 can mediate PD-L1 ubiquitination-dependent proteolysis and lysosomal degradation simultaneously [46]. Wang et al.‘s research also supported the idea that CMTM6 and Huntingtin-interacting protein 1-related protein (HIP1R) participate in the lysosomal degradation of PD-L1. Overall, CMTM6 is a promising target for immunotherapy, as indicated by these findings [47]. (Fig. 2)

The ubiquitination of PD-L1 is also associated with its trafficking. Ding’s team found that sodium-glucose cotransporter 2 (SGLT2) colocalizes with PD-L1 in the cell membrane and circulating endosomes, preventing proteasome-mediated PD-L1 degradation [48]. Yu et al. discovered that MIB E3 ubiquitin protein ligase 2 (MIB2) catalyzes the nonproteolytic K63-linked polyubiquitination of PD-L1, facilitating its transport of PD-L1 from the trans-Golgi network (TGN) to the membrane through RAS-associated binding 8-mediated (RAB8-mediated) exocytosis [49]. (Fig. 2)

Noncoding RNAs significantly contribute to the ubiquitination of PD-L1. Knockdown of circ-0000512 enhances PD-L1 ubiquitination in triple-negative breast cancer (TNBC) cells by inhibiting CMTM6 [50]. The circular RNA hsa_circ_0067842 interacts with HuR, improving the stability of CMTM6 by influencing nuclear translocation. CMTM6, in turn, regulates the ubiquitination of PD-L1 and inhibits its degradation [51]. (Fig. 2)

### Other PTMs inhibiting PD-L1 expression and function

**Fig. 3:**
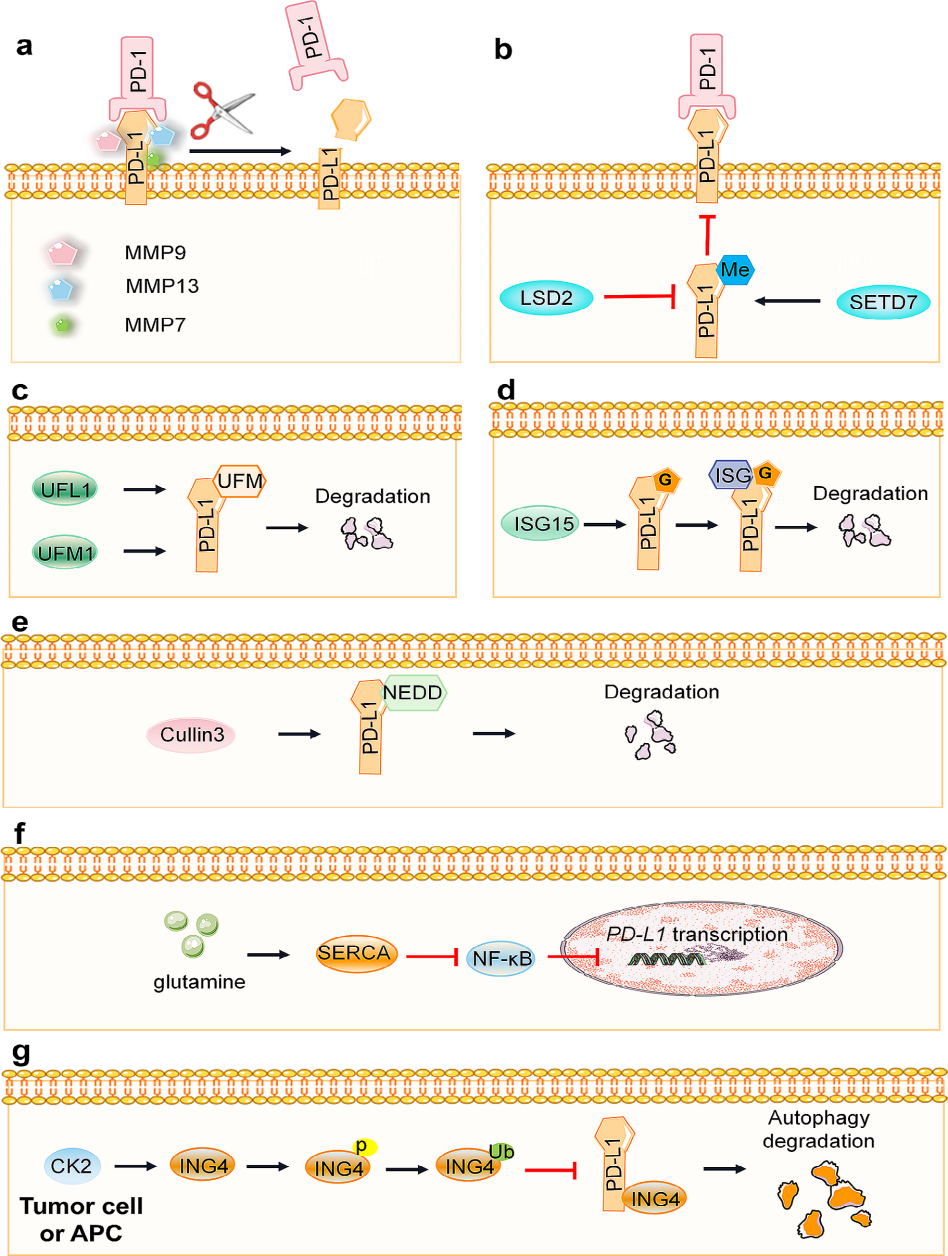
Negative regulatory pathways of PD-L1 mediated by other posttranslational modifications. **a** MMP-7, MMP-9, and MMP-13 cleave the PD-1 binding domain of PD-L1, inhibiting T-cell apoptosis. **b** PD-L1 proteins undergo methylation by SET7 and demethylation by LSD2. **c** UFL1 or UFM1 enhances the UFMylation of PD-L1. **d** ISG15 associates with glycosylated PD-L1, promoting its ISGylation and accelerating the glycosylation-mediated degradation of PD-L1. **e** Cullin3 promotes NEDDylation, which contributes to the degradation of the PD-L1 protein. **f** GSH upregulates SERCA activity, suppressing the NF-κB signaling cascade and consequently the transcription of PD-L1. **g** CK2 induces phosphorylation of ING4, leading to the activation of ING4 and subsequent inhibition of the proteolytic degradation of PD-L1. The black arrows indicate positive regulation, and the red arrows indicate negative regulation

Ectodomain shedding is a posttranslational modification involving the degradation of extracellular matrix components. Matrix metalloproteinases (MMPs) and disintegrin and metalloproteinases (ADAMs) convert transmembrane molecules into soluble forms in this process [52, 53]. The proteolytic cleavage of PD-L1 is attributed to the release of MMP-13 from fibroblasts. MMP-9 and MMP-13 have been identified as enzymes capable of cleaving the PD-1 binding domain of PD-L1, consequently inhibiting T-cell apoptosis [54]. Hira-Miyazawa et al. further confirmed that purified PD-L1 can undergo degradation by MMP-13 and MMP-7. A specific inhibitor of MMP-13 (CL82198) significantly restored the expression of PD-L1, providing additional evidence for the pivotal role of MMP-13 in the shedding/cleavage of PD-L1 [55]. Known as an effective inhibitor of MMPs, HE4 was investigated by Rowswell-Turner RB et al., who revealed its ability to inhibit MMP2, 9, and 13. This inhibition resulted in a significant increase in PD-L1 expression in both tumors and macrophages, and this effect was observed posttranscriptionally [56]. (Fig. 3a)

Protein methylation is a prevalent modification that can occur on both histone and nonhistone proteins and typically affects arginine and lysine residues. Arginine methylation, a common posttranslational modification, involves the addition of methyl groups to arginine residues, altering the protein’s interactions with binding partners or regulating its activity [57]. Nonhistone methylation often participates in signal transduction, with many instances linked to cancer progression [58]. In a study by Huang et al., six monomethylation sites (K75, K89, K105, R113, K162, and R212) were identified on PD-L1 through mass spectrometry (MS) analysis. Interestingly, the K162R variant was the only variant demonstrated to enhance the engagement of PD-1/PD-L1. PD-L1 methylation at K162 restricted the interaction between PD-L1 and PD-1 [22]. SET domain containing lysine methyltransferase 7 (SETD7) catalyzes the methylation of PD-L1 at the K162 site, and this modification can be reversed by LSD2. Therefore, hypermethylation of PD-L1 has been identified as a key mechanism of resistance to PD-L1 therapy [22]. (Fig. 3b)

PD-L1 is also targeted by ubiquitin-fold modifier 1 (UFM1) modification (UFMylation) [59]. UFM1 is initially synthesized in its precursor form. Upon cleavage by UFSP1 or UFSP2, UFM1-G83 is activated. This activated form is processed by the specific E1-like activating enzyme UBA5 and then transferred to the E2-like binding enzyme UFC1. The final step involves the collaboration of UFC1 with the E3-like ligase UFL1 [60]. Silencing either UFL1 or UFM1 to suppress the UFMylation of PD-L1 can lead to its stabilization in various human and mouse cancer cells, which in turn disrupts anticancer immunity both in vitro and in mice [59]. (Fig. 3c)

Interferon-stimulating gene 15 (ISG15) modification (ISGylation) is a process similar to ubiquitination. During ISGylation, the target protein binds to ISG15, modifying the target protein. Subsequently, the modified target protein and ISG15 are separated by ISG15 depolymerase, and the separated ISG15 can be recycled [61]. ISG15 induces ISG modification and PD-L1 protein instability, thereby improving targeted immunotherapy targeting PD-L1 and inhibiting the growth of lung adenocarcinoma in vivo. Additionally, ISG15 enhances K48-linked ubiquitin modification of PD-L1, ultimately promoting the degradation of glycosylated PD-L1 through the proteasome pathway [62]. (Fig. 3d)

Neural precursor cell-expressed developmentally downregulated 8 (NEDD8) modification (NEDDylation), a process similar to ubiquitination, involves the coupling of the active ubiquitin-like protein NEDD8 with the scaffold Cullin protein by the E3 Cullin-RING ligase (CRL) [63]. Pevonedistat (MLN4924, TAK924) is a small molecule inhibitor of NEDD8. Pevonedistat blocks the degradation of the PD-L1 protein by inhibiting Cullin3 activity [64, 65], increasing the levels of PD-L1 mRNA and protein in a dose- and time-dependent manner [66]. (Fig. 3e)

S-glutathionylation is a common form of cysteine (Cys or C) modification that involves the reversible formation of mixed disulfide bonds with glutathione (GSH). According to Byun JK et al., inhibiting glutamine utilization increases PD-L1 levels in cancer cells, thereby inactivating cocultured T cells [67]. Restricting glutamine metabolism in cancer cells can impair sarcoplasmic/endoplasmic reticulum calcium ATPase (SERCA) activity by reducing S-glutathionylation due to low glutathione levels. This activates the calcium/NF-κB signaling cascade, ultimately leading to the transcriptional activation of PD-L1 [67]. (Fig. 3f)

Autophagy serves as the primary intracellular degradation system, ushering cytoplasmic substances into lysosomes for breakdown and generating new components and energy for cellular homeostasis [68]. Gou et al. demonstrated that growth inhibitory factor 4 (ING4) induces autophagic degradation of PD-L1, suppressing immune escape in NSCLC cells by enhancing T-cell activity. Additionally, casein kinase 2 (CK2) phosphorylates ING4 at S150, promoting its ubiquitination and degradation via the JFK ubiquitin ligase. Conversely, CK2 gene knockout strengthens ING4 protein stability and augments T-cell activity [69]. (Fig. 3g)

## Preclinical study of PTMs promoting PD-L1 expression and function

### Deubiquitination of PD-L1 upregulates its expression by enhancing protein stability

**Fig. 4:**
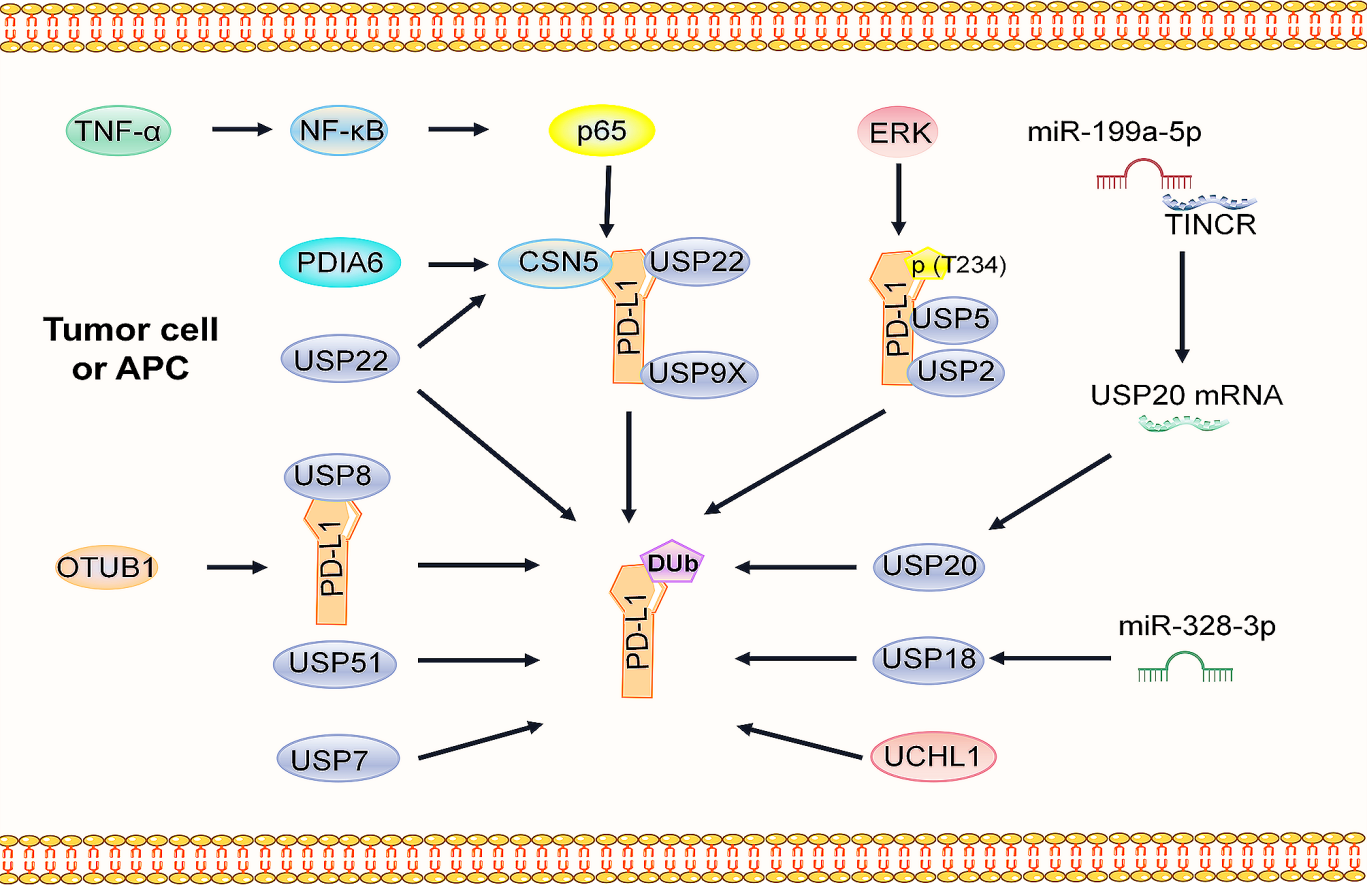
Regulatory pathways of PD-L1 via deubiquitination. The deubiquitination of PD-L1 involves a family of ubiquitin-specific proteases (USPs), namely, USP2, USP5, USP7, USP8, USP9X, USP20, USP22, USP28, and USP51, along with CSN5, UCHL1, OTUB1, and microRNAs (miR-199a-5p and miR-328-3p). Positive regulatory interactions are denoted by black arrows

Deubiquitination is a process catalyzed by deubiquitination enzymes (DUBs), which reverse ubiquitination by removing ubiquitin molecules from ubiquitinated proteins [70, 71]. In contrast to ubiquitination, the deubiquitination of PD-L1 can enhance the stability of the protein. COP9 signaling body 5 (CSN5) plays a crucial role in the CSN complex, contributing to tumor immune escape by inducing the deubiquitination of PD-L1 [72]. Lim et al. reported that TNF-α secreted by macrophages in breast cancer (BC) impacts PD-L1 expression at the translational level. TNF-α induces the expression of CSN5 and CSN2 by activating p65 of NF-κB [73]. Subsequently, CSN5 binds to the C-terminus of PD-L1 and deubiquitinates it, thereby enhancing its stability. Although the MPN domain of CSN5 does not interact with PD-L1, disruption of the MPN domain affects the CSN5-mediated deubiquitination of PD-L1 and protein stability [73]. Protein disulfide isomerase A (PDIA)6 might upregulate the expression of CSN5 by regulating the formation of disulfide bonds in CSN5, increasing the stability of PD-L1 through deubiquitination in pancreatic cancer cells [74, 75]. (Fig. 4)

USPs have been identified as novel deubiquitinases of PD-L1 in multiple cancers. USP22 specifically targets the C-terminus of PD-L1, leading to its deubiquitination and stabilization in liver cancer cells [76]. Additionally, USP22 enhances the stability of CSN5 both by deubiquitination and by directly regulating PD-L1 deubiquitination in NSCLC. This process enhances PD-L1 stability by removing the K6, K11, K27, K29, and K33 residues that bind to PD-L1 [77]. USP9X binds to PD-L1, inducing its deubiquitination and stabilizing protein expression in OSCC [78]. Thr288, Arg292, and Asp293 on USP2 regulate its binding to PD-L1, uncoupling the K48-linked residue on lysine 270 of PD-L1 to increase PD-L1 abundance. Deletion of USP2 leads to the degradation of ER-related PD-L1, which weakens the binding of PD-L1 to PD-1 and renders cancer cells susceptible to T-cell-mediated cytotoxicity [79]. USP51 enhances the stability of the PD-L1 protein by removing polyubiquitination, promoting chemotherapy resistance in NSCLC cells [80]. In pancreatic cancer, USP8 inhibits the ubiquitination-regulated proteasome degradation pathway by positively interacting with PD-L1 and upregulating its expression [81]. USP7 mediates the ubiquitination of PD-L1 and inhibits its degradation [82]. Additionally, UCHL1 promotes PD-L1 deubiquitination and upregulates its expression in NSCLC [83]. OTUB1 interacts with and removes K48-linked ubiquitin strands in the PD-L1 cytoplasmic domain via a process mediated by deubiquitinase activity, preventing PD-L1 degradation through the ER-associated degradation (ERAD) pathway [84]. (Fig. 4)

Noncoding RNAs are also known to be involved in regulating PD-L1 deubiquitination. The long noncoding RNA TINCR functions as a sponge of miR-199a-5p, enhancing the stability of USP20 mRNA through a competitive endogenous RNA mechanism. This causes PD-L1 to become ubiquitinated and increases its protein abundance [85]. . Zheng’s team found that the smoking-related lncRNA BCCE4 mutation rs62483508 G > A can disrupt the binding site of miR-328-3p, reducing the expression of USP18 and weakening the interaction between PD-L1/PD-1 to strengthen antitumor immune responsiveness in bladder tumors [86]. (Fig. 4)

Small ubiquitin-like modifier (SUMO) modification (SUMOylation) is correlated with deubiquitination [87]. Ma X et al. discovered that the E3 SUMO ligase tripartite motif-containing protein 28 (TRIM28) can stabilize the PD-L1 protein by inhibiting PD-L1 ubiquitination and promoting its SUMOylation in gastric cancer cells [88].

### Glycosylation of PD-L1 promotes its protein expression and function

Glycosylation is a crucial modification that can significantly impact protein formation, function, and interactions with other proteins. The process of glycosylation involves the formation of glycoproteins with specific oligosaccharide chains in the ER, facilitated by various glycosyltransferases and glycosidases. Subsequently, glycoproteins move from the Cis surface to the Golgi body, where they undergo a series of ordered processing and modifications. N-linked glycosylation and O-linked glycosylation. N-linked glycosylation attaches a sugar chain to the -NH2 group of an asparagine residue, while O-linked glycosylation links a sugar chain to the oxygen of -OH groups in serine, threonine, or hydroxylysine residues of a polypeptide [89]. Hypoxia and abnormal glucose metabolism are known to alter protein glycosylation patterns in the tumor microenvironment. Notably, PD-L1 is highly glycosylated in most cells expressing it, while the unglycosylated form tends to have lower expression levels [18].

#### N-glycosylation of PD-L1 positively regulates its protein stability and interaction with PD-1

**Fig. 5:**
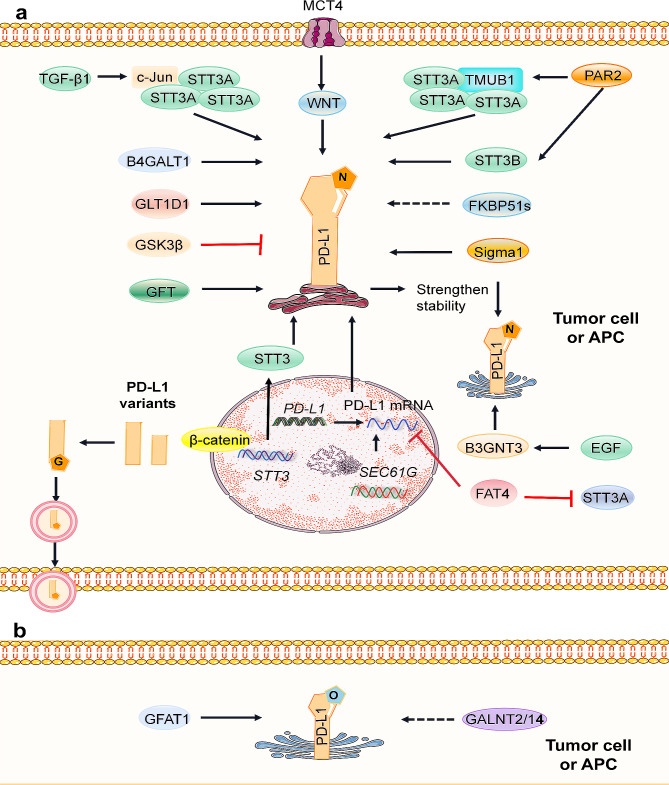
Regulatory pathways of PD-L1 through glycosylation. **a** Sigma1 promotes N-glycosylation in both the ER and Golgi, and FKBP51 s also augment N-glycosylation of PD-L1 in the ER. EGF upregulates B3GNT3, facilitating the glycosylation of PD-L1 in the Golgi apparatus. Conversely, GSK3β inhibits PD-L1 glycosylation. GLT1D1, GFT, and B4GALT1 promote the N-glycosylation of PD-L1. β-catenin induces N-glycosyltransferase STT3 transcription, stabilizing the oligosaccharide chains of PD-L1 in the ER and upregulating PD-L1. PAR2 activates the N-glycosyltransferases STT3A and STT3B, enhancing the glycosylation of PD-L1. TMUB1 enhances the N-glycosylation and stability of PD-L1 by recruiting STT3A. TGF-β1 activates the c-Jun/STT3A signaling pathway, promoting N-glycosylation of PD-L1. At the transcriptional level, FAT4 reduces PD-L1 mRNA expression and downregulates STT3A through β-catenin, resulting in abnormal glycosylation of PD-L1. SEC61G induces PD-L1 translation and N-glycosylation. MCT4 stabilizes PD-L1 by promoting its glycosylation through the classical WNT pathway. Notably, secreted PD-L1 splicing variants exist, with only those possessing N-linked glycosylation sites exhibiting stable secretion. **b** GALNT2/14 and GFAT1 potentially increase the O-glycosylation of PD-L1. The black arrows denote positive regulation, while the red arrows indicate negative regulation

Glycosylation of PD-L1 plays a crucial role in promoting its protein stability. Specifically, the N192, N200, and N219 sites on the PD-L1 protein hinder the interaction between GSK3β and PD-L1 [90]. The inhibition of GSK3β facilitates the glycosylation of PD-L1 in breast cancer, preventing its degradation by the 26 S proteasome [91]. Sigma1 has been implicated in regulating the glycosylation of newly synthesized PD-L1 in the ER and Golgi compartments to promote the expression of PD-L1 [91]. FK506 binding protein 51 s (FKBP51 s), which are specifically expressed in glioblastoma, promote the glycosylation of PD-L1 in the ER and upregulate its expression on cell membranes [92]. Glycosyltransferase 1 domain 1 (GLT1D1) enhances the stability of PD-L1 through N-glycosylation, promoting immunosuppression and tumor growth [93]. The GDP-fucose transporter (GFT) is a critical molecule involved in fucosylation of PD-L1. Knockout of the GFT gene SLC35C1 significantly decreases PD-L1 fucosylation, leading to increased ubiquitination of PD-L1 [94]. Beta-1,4-galactosyltransferase 1 (B4GALT1) directly mediates the N-glycosylation of PD-L1, preventing its degradation. Inhibition of B4GALT1 increases the abundance and activity of CD8^+^ T cells, enhancing antitumor immunity against PD-1 therapy in vivo [95]. In breast cancer tumor stem cells, the enrichment of PD-L1 is considered crucial for tumor stem cell immune escape. The mechanism involves β-catenin inducing the transcription of the N-glycosyltransferase STT3 to promote the oligoglycosylation of PD-L1 in the ER and upregulate PD-L1 expression [96]. PD-L1 enhances its stability by activating the N-glycosyltransferases STT3A and STT3B through PAR2 [97]. Additionally, TMUB1 enhances the N-glycosylation and stability of PD-L1 by recruiting STT3A, which promotes PD-L1 maturation and facilitates tumor immune escape [39]. TGF-β1 activates the c-Jun/STT3A signaling pathway, promoting the N-glycosylation of PD-L1 [98]. FAT atypical cadherin-4 (FAT4) overexpression not only reduces PD-L1 mRNA expression but also inhibits STT3A by promoting β-catenin degradation. This triggers aberrant glycosylation of PD-L1, causing its accumulation in the ER and degradation by ubiquitin-dependent pathways [99]. The gene SEC61G, located adjacent to the EGFR chromosome, promotes the translocation of immune checkpoint ligands (PD-L1, PVR, and PD-L2) to the ER, facilitating their glycosylation, stability, and membrane presentation [100]. Monocarboxylate transporter 4 (MCT4) has been found to promote the glycosylation of PD-L1 through the classical WNT pathway, stabilizing PD-L1. The high coexpression of MCT4 and PD-L1 suggests a more effective target for treating TNBC, potentially improving the immune checkpoint treatment of TNBC [101]. (Fig. 5)

PD-L1 glycosylation is essential for the interaction of PD-L1 with PD-1. While the signaling of costimulatory molecules can function effectively without glycosylation, the signaling of coinhibitory molecules, including PD-L1, requires glycosylation, particularly N-linked glycosylation [90]. Furthermore, activation of the EGF/EGFR signaling pathway has been shown to upregulate beta-1,3-N-acetylglucosaminyltransferase 3 (B3GNT3), promoting the glycosylation of poly-N-acetyllactosamine at the N192 and N200 sites of PD-L1 in the Golgi apparatus. This enhanced glycosylation, mediated by B3GNT3, increases the affinity of PD-L1 for binding to PD-1 [90]. Molecular dynamics simulations of the PD-L1/PD-1 interaction with N-glycans suggest that N-glycosylation of the PD-L1 N219 site may influence the interaction with PD-1 [18]. (Fig. 5)

The glycosylation of PD-L1 appears to be involved in the promotion of tumor metastasis. Erlichman and his team reported that PD-L1 activates STAT1 and STAT3 to promote breast cancer cell metastasis both in vitro and in vivo and that PD-L1 is required for N-glycosylation at the N219 site [102]. In addition, the glycosylation sites N192 and N200 (depending on cell type) contribute to the autonomous cell migration function of PD-L1 in vitro [102]. (Fig. 5)

The glycosylation of secreted PD-L1 variants has been implicated in drug resistance to PD-L1 antibodies. Gong et al. identified five secreted PD-L1 splicing variants in patients resistant to anti-PD-L1 antibodies: PD-L1 v174, PD-L1 v178, PD-L1 v229, PD-L1 v242, and PD-L1 v265. Among these variants, PD-L1 v242 and PD-L1 v229 contain three N-glycosylation sites (N192, N200, and N219), which contribute to the stabilization of PD-L1, allowing it to be stably secreted and induce resistance to anti-PD-L1 antibodies [103]. Conversely, PD-L1 v178 lacks N-glycosylation sites, making it unstable and poorly secreted. As a splicing variant of PD-L1, PD-L1-vInt4 functions as bait in anti-PD-L1 antibody therapy, further contributing to drug resistance [104]. This finding sheds light on a novel mechanism of drug resistance against anti-PD-L1 antibodies. (Fig. 5)

#### O-linked glycosylation of PD-L1 may be related to its expression

GALNT2/14, which are polypeptide N-acetyl glucosaminyl transferase 2/14, play a role in initiating mucin O-glycosylation in the Golgi apparatus. Research has demonstrated a positive correlation between the expression of GALNT2/14 and that of PD-L1 [105]. However, conflicting studies have suggested that the stability of the PD-L1 protein might not be dependent on O-linked glycosylation [106]. Chen et al. reported that although inhibiting L-glutamine: D-fructose-6-phosphate aminotransferase 1 (GFAT1) reduces overall protein O-GlcNAcylation, it does not seem to affect the stability of PD-L1. The increase in PD-L1 protein degradation is attributed to the decrease in N-linked glycosylation, even though other mechanisms cannot be ruled out [106]. (Fig. 5)

### Other PTMs inhibiting PD-L1 expression and function

**Fig. 6:**
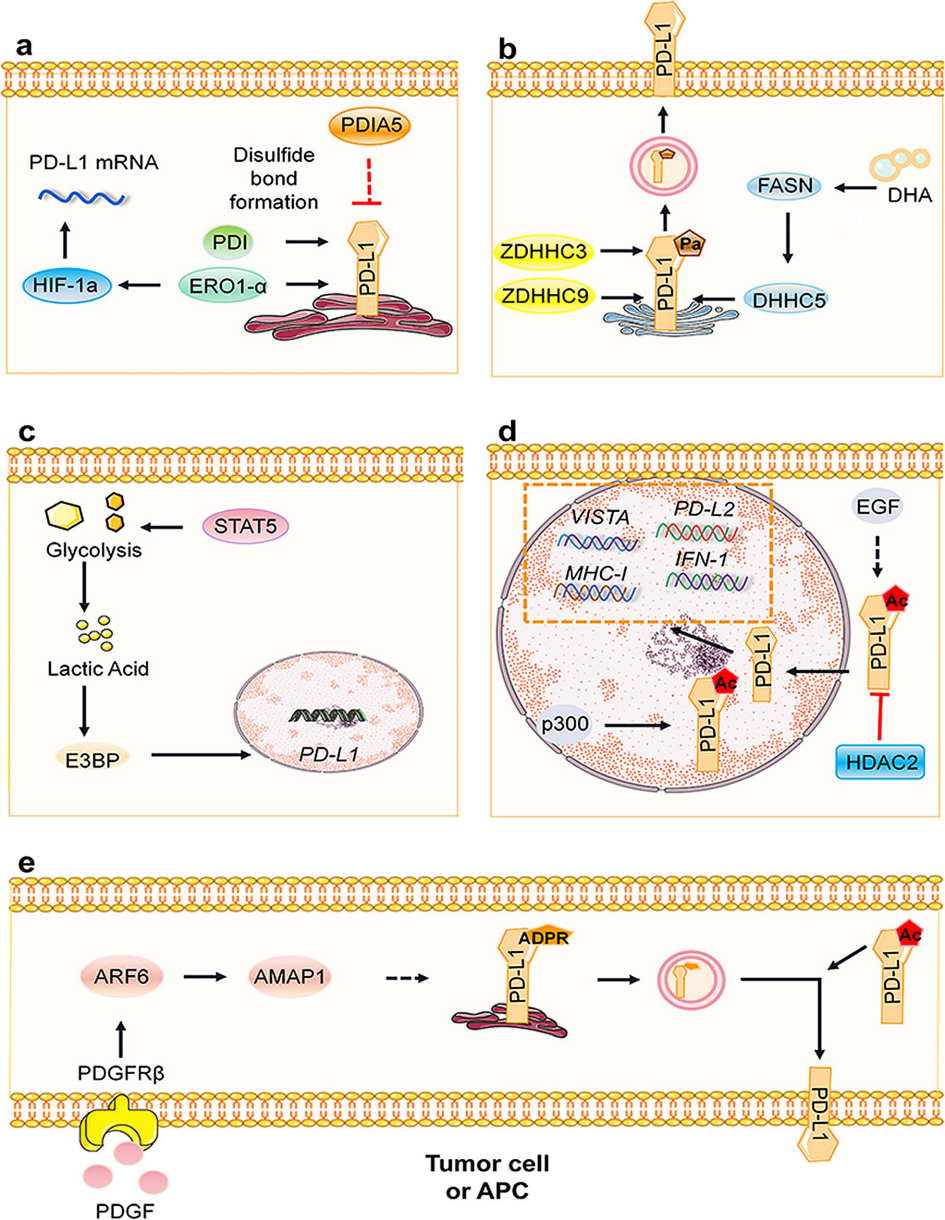
Positive regulatory pathways of PD-L1 mediated by other posttranslational modifications. **a** PDIA5 appears to exert a negative regulatory effect on PD-L1, while ERO1-a enhances PD-L1 expression by facilitating the proper formation of disulfide bonds in PD-L1. ERO1-α additionally upregulates HIF-1a protein, resulting in increased PD-L1 mRNA and protein levels. **b** S-palmitoylation occurs within the Golgi apparatus. ZDHHC9, DHHC3, DHHC5, and FASN have been identified as promoters of PD-L1 palmitoylation and thereby contribute to the stabilization of the PD-L1 protein. Conversely, DHA inhibits FASN, thereby suppressing the palmitoylation of PD-L1. **c** STAT5, which promotes glycolysis, leads to lactic acid accumulation, subsequently facilitating E3BP nuclear translocation and histone lactylation, culminating in the induction of PD-L1 transcription. **d** HDAC2 facilitates nuclear translocation through PD-L1 deacetylation, whereas p300 promotes acetylation, enhancing its interaction with TRAPPC4 and facilitating PD-L1 recycling to the membrane. **e** PDGF/ARF6/AMAP1 enhances the recycling of PD-L1 to the membrane by augmenting the ADP-ribosylation of PD-L1. The black arrows denote positive regulation, while the red arrows signify negative regulation

Secreted and membrane proteins often contain numerous disulfide bonds formed by the oxidation of two Cys residues, which are crucial for their structural stability and function. Incorrect disulfide bond formation can cause protein misfolding in the ER, triggering the unfolded protein response (UPR) to manage protein folding [107]. ERO1-α, an ER oxidase often overexpressed in tumors, works with protein disulfide isomerase (PDI) to form disulfide bonds. Studies by Tanaka et al. have shown that ERO1-α enhances PD-L1 expression by facilitating the folding of oxidized proteins in PD-L1 [108]. Chen et al. reported that silencing PDIA5 in human glioma cells upregulates PD-L1 expression, suggesting that PDIA5, by modifying disulfide bonds and activating the UPR, may influence PD-L1 expression, although the exact mechanisms involved are unexplored [109]. (Fig. 6a)

Palmitoylation, a lipid modification, is essential for regulating membrane proteins and includes S-palmitoylation, N-palmitoylation, and O-palmitoylation [110]. S-palmitoylation involves attaching a 16-carbon fatty acid palmitate to Cys residues via an unstable covalent bond, which is typically catalyzed by DHHC palmitoyl transferase [110, 111]. This is a pivotal modification in several cancer-related proteins, including PD-L1, where C272 palmitoylation helps stabilize the protein, thus protecting tumor cells from being eliminated by T cells. In breast cancer, ZDHHC9 enhances PD-L1 stability through palmitoylation [112, 113], and ZDHHC9 deficiency in lung cancer prevents PD-L1 degradation, enhancing the effectiveness of anti-PD-L1 immunotherapy [115]. Similarly, ZDHHC3 increases PD-L1 palmitoylation at C272 in colorectal and pancreatic cancer models, reducing its degradation [116]. Shahid et al. reported that fatty acid synthase (FASN) in cisplatin-resistant bladder cancer cells enhances PD-L1 expression by regulating palmitate formation [117]. Moreover, DHA downregulates FASN, inhibits DHHC5, and promotes PD-L1 degradation [41]. Addressing PD-L1 palmitoylation may be an effective way to counteract tumor immune evasion strategies. (Fig. 6b)

Succinylation correlates with increased PD-L1 expression in prostate cancer, suggesting its significant role in regulating PD-L1 levels [118–121]. Additionally, lactic acid, a precursor of histone lysine modifications, is linked to glycolytic gene activation by STAT5 in AML, leading to increased PD-L1 transcription via enhanced histone lactylation and nuclear translocation of E3BP [122, 123]. These modifications reveal intricate connections between metabolic processes and immune regulation in cancer. (Fig. 6c)

Protein lysine acetylation, which is reversible via lysine acetylases (KATs), influences protein stability and localization [124, 125]. Recent findings have shown that nuclear PD-L1, which is acetylated at K263 by p300 and deacetylated by HDAC2, acts as a transcription factor that alters gene expression related to antigen presentation and inflammatory pathways, affecting cytotoxic T lymphocyte activity and tumor immune evasion [126]. Nuclear PD-L1 also upregulates other immune checkpoint genes and angiogenesis markers in breast cancer. EGF enhances PD-L1 acetylation [33], while VPA increases PD-L1 recycling to the membrane, highlighting complex regulatory mechanisms [127]. (Fig. 6d)

ADP-ribosylation is a dynamic, reversible posttranslational modification that involves the attachment of an ADP-ribose group to proteins, affecting their degradation and vesicle transport between organelles [128–132]. This modification is initiated by NAD^+^ cleavage, leading to either mono- or multi-ADP-ribosylation. Hashimoto et al. reported that PDGF binding to its receptor, PDGFRβ, activates ADP-ribosylation factors such as ARF6 and AMAP1, promoting PD-L1 recycling to the cell membrane; silencing these factors reduces PD-L1 surface expression, illustrating the role of ADP-ribosylation in vesicle transport [130, 131]. (Fig. 6e)

## Preclinical study of PTMs regulating PD-1 expression and function

### Phosphorylation of PD-1 affects its immunosuppressive effect

**Fig. 7:**
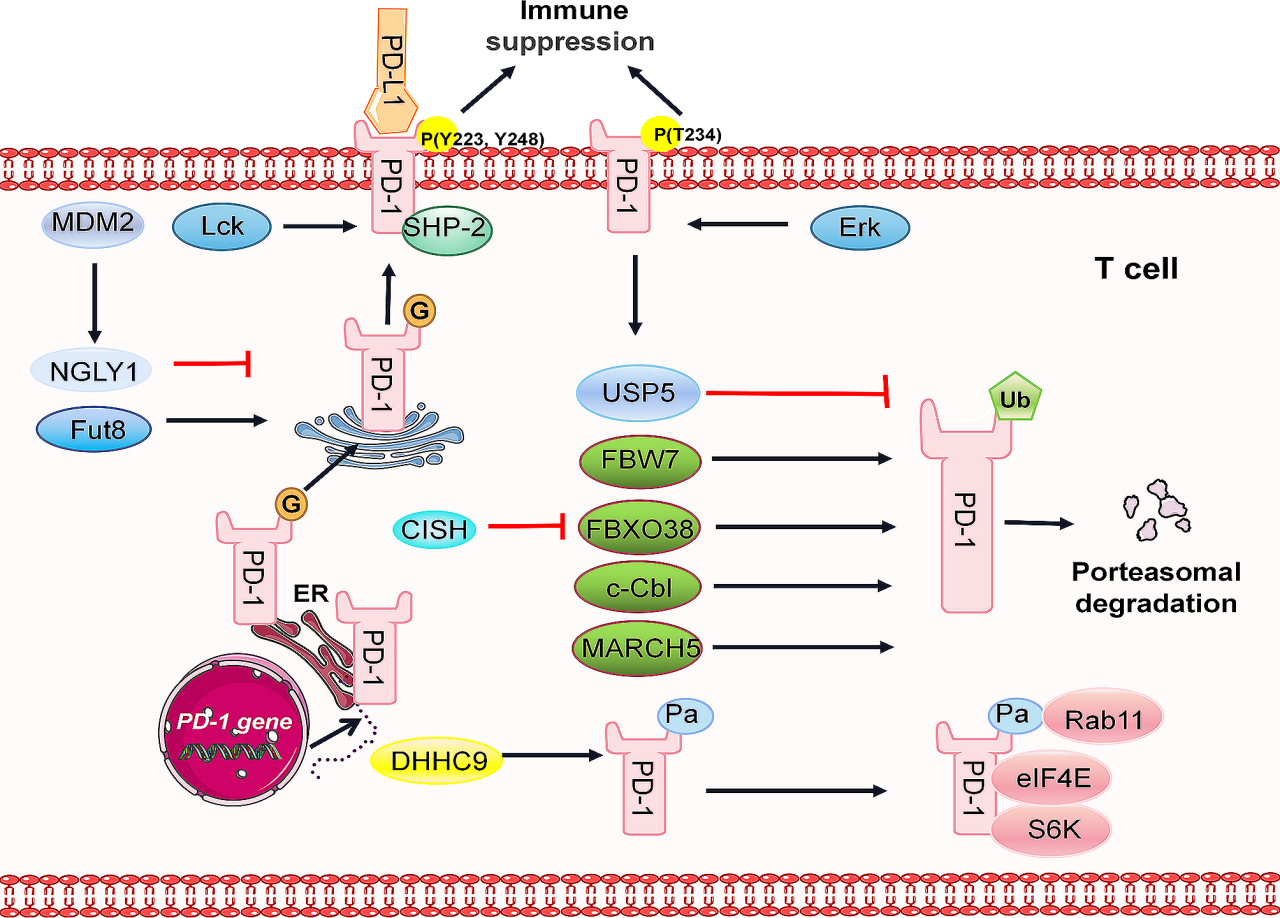
Posttranslational modification of PD-1. As a transmembrane protein, PD-1 undergoes intricate posttranslational modifications. The primary site of focus for PD-1 is within the Golgi apparatus. Fut8 plays a pivotal role in promoting the core structure of the PD-1 protein, thereby contributing to the stabilization of PD-1. Upon binding to PD-L1, the intracellular domain of PD-1 undergoes phosphorylation, recruiting SHP2-2 and subsequently initiating immunosuppressive signaling. Lck enhances the phosphorylation of PD-1, intensifying its downstream effects. IL-2 promotes the transcription of FBXO38, which, in turn, binds to the cytoplasmic region of PD-1, facilitating polyubiquitination and subsequent proteasome-mediated degradation. MARCH5, c-Cbl, and FBW7 are also implicated in promoting PD-1 ubiquitination. MDM2 facilitates the interaction between NGLY1 and PD-1, leading to the deglycosylation of PD-1. Furthermore, DHHC9 promoted the palmitoylation of PD-1 to enhance its interaction with Rab11. Inhibition of palmitoylation diminishes the transport of PD-1 to the recycling endosome, promoting its degradation in the lysosome. This process is also associated with a notable enhancement in the interaction between PD-1 and mTOR signal effector proteins (S6K and eIF4E). The black arrows indicate positive regulatory pathways, while the red arrows indicate negative regulatory pathways

Tyrosine phosphorylation within the PD-1 ITSM domain is a pivotal step in the activation of downstream immunosuppressive pathways. Upon interaction between PD-1 and PD-L1, phosphorylation occurs at the PD-1 ITIM (Y223) and ITSM (Y248). The phosphorylation of ITSM results in the recruitment of protein tyrosine phosphatase 2 (SHP2), which subsequently dephosphorylates the ζ chains and ζ chain-related tyrosine kinase 70 (ZAP70) within CD28 and the T-cell receptor (TCR)/CD3 complex. This inhibition affects the downstream PLCγ1, PI3K/AKT, and ERK1/2 signaling pathways, leading to reduced IL-2 secretion and glucose metabolism. Consequently, T-cell function is further inactivated, playing a negative role in immune regulation [27]. Hui et al. reported that CD28 and PD-1 cluster briefly and concentrically near the TCR when PD-1 on T cells binds to PD-L1. The TCR phosphorylation kinase Lck effectively phosphorylates PD-1, while SHP2 dephosphorylates PD-1, rendering PD-1 unstable. In the absence of SHP2, SHP1 can assume its role [133]. Similarly, upon binding of PD-L1 to PD-1 on B cells, tyrosine in the PD-1 ITSM domain undergoes phosphorylation [134]. Furthermore, ERK can phosphorylate the T234 site of PD-1, subsequently promoting the interaction between PD-1 and USP5, which results in deubiquitination and enhanced stability of PD-1 [135]. (Fig. 7)

### Ubiquitination of PD-1 mediates its degradation and regulates the antitumor immunity of T cells

Factors within the tumor microenvironment can induce high expression of the inhibitory receptor PD-1 on functional T cells. However, there is limited understanding of the degradation mechanism of PD-1. FBXO38 is recognized as the E3 ligase responsible for PD-1, directly targeting the PD-1 cytoplasmic domain and mediating its K48-linked polyubiquitination, followed by proteasome degradation [13]. IL-2 treatment significantly enhances the transcription of F-box protein 38 (Fbxo38), reducing PD-1 levels and boosting anticancer activity in mice [13]. Lv et al. elucidated that cytokine-inducible SH2 domain-containing protein (CISH) promotes PD-1 expression by inhibiting FBXO38 expression, suggesting novel strategies to enhance CAR-T-cell therapeutic efficacy by inhibiting CISH [136]. The C-terminus of c-Cbl interacts with the cytoplasmic tail of PD-1 and destabilizes PD-1 through ubiquitination-proteasome degradation in mouse colorectal cancer [137]. Additionally, F-box and wd repeat domain containing 7 (FBW7) has been shown to promote the ubiquitination of PD-1 and subsequent proteasome hydrolysis [12]. Recently, Wu et al. demonstrated that ubiquitination and breakdown of PD-1 require elimination of N-linked glycosylation and identified MDM2 as an E3 ligase for deglycosylation of PD-1. These enzymes facilitate the interaction between glycosylated PD-1 and N-glycanase 1 (NGLY1), leading to further deglycosylation of PD-1 catalyzed by NGLY1 [138]. These preclinical studies suggest that the ubiquitination of PD-1 is expected to become a new focus in the development of anticancer medications [139]. (Fig. 7)

### N-glycosylation of PD-1 impacts its protein expression and interaction with PD-L1

The attachment of PD-1 to its ligands is dependent on the N49, N58, N74, and N116 glycosylation sites located in the PD-1 IgV domain [11]. Core fucosylation at N49 and N74 regulates PD-1 expression. The inhibition of core fucosylation through the use of 2-fluoro-L-fucose (2 F-Fuc), which targets the fucosyltransferase Fut8, results in decreased PD-1 expression and T-cell activation [140]. (Fig. 7)

### Palmitoylation of PD-1 upregulates its expression and interaction with mTOR signaling effectors

Palmitoylation of PD-1 plays a crucial role in inhibiting lysosomal degradation, thereby stabilizing the protein. Yao et al. reported that DHHC9 promotes the palmitoylation of PD-1, leading to interaction with Rab11, which is a pivotal molecule facilitating the transport of cargo proteins to recycled endosomes [141]. Blocking palmitoylation reduces PD-1 transport to recycled endosomes and enhances lysosomal degradation. Intriguingly, PD-1 palmitoylation significantly enhances the interaction between PD-1 and mTOR signaling effectors (S6K and eIF4E), activating mTOR signaling and promoting tumor growth [141]. (Fig. 7)

## Therapeutic prospects and clinical transformation of PD-1/PD-L1 PTMs

**Fig. 8:**
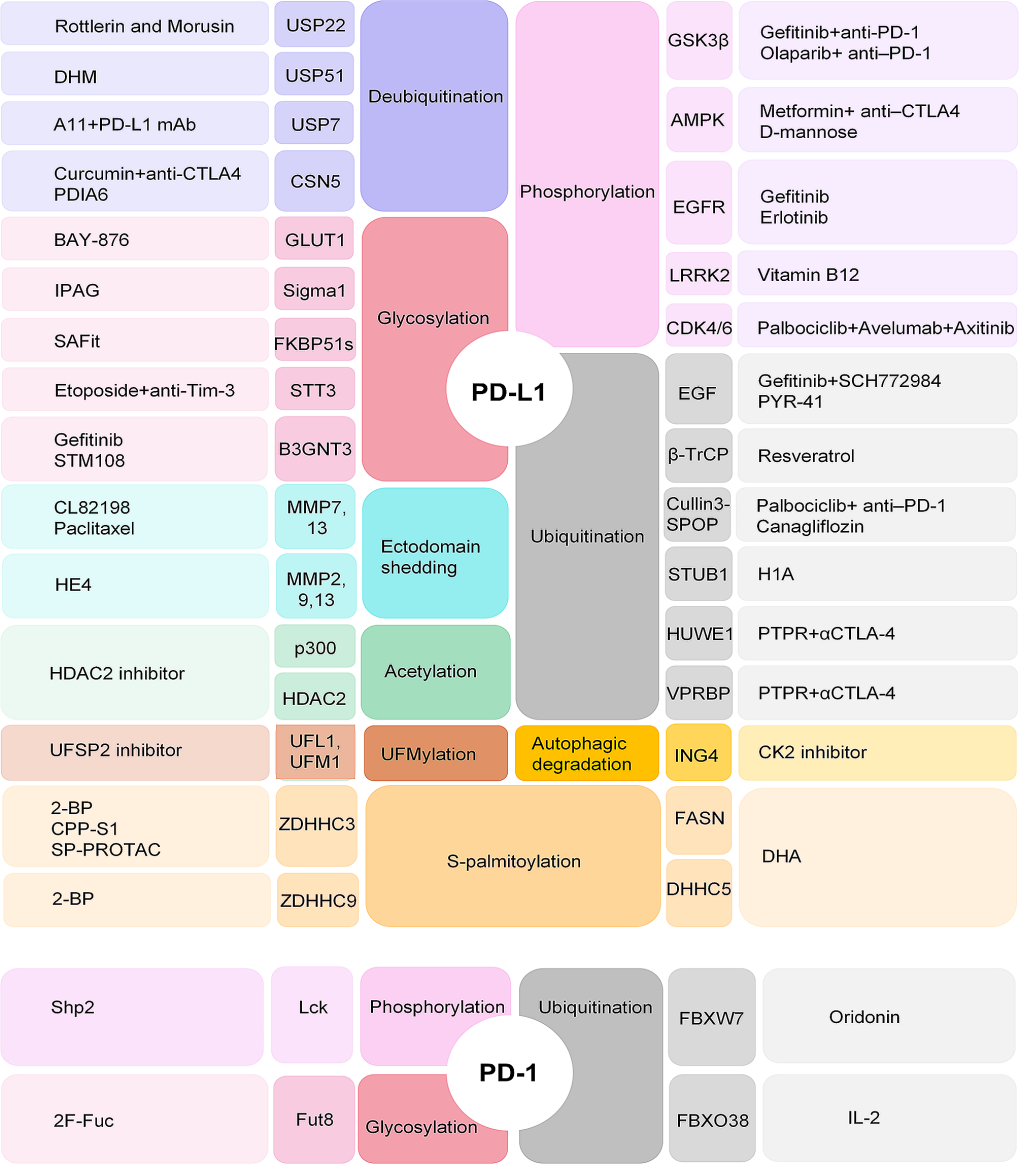
Regulatory networks and corresponding therapeutic interventions targeting PD-1/PD-L1 posttranslational modifications. The figure illustrates various therapies targeting PD-L1 and PD-1 post-translational modifications. The colored regions—purple, red, blue, green, brown, pink, gray, orange, and yellow—correspond sequentially to deubiquitination, glycosylation, ectodomain shedding, acetylation, UFMylation, phosphorylation, ubiquitination, autophagy degradation, and S-palmitoylation modifications. Adjacent to each colored region, the outer grids display the related molecules and potential therapeutic drugs targeting these specific post-translational modifications

Building on foundational research into PTMs of PD-1/PD-L1 that regulate their expression and function, researchers have developed targeted therapies tested in cell and mouse models (Table 1; Fig. 8). Currently, these promising results are moving toward clinical applications, with multiple treatment regimens involving PTM-targeting drugs and immune checkpoint inhibitors actively progressing through clinical trials. These efforts aim to validate and expand the use of these innovative therapies in clinical settings (Table 2).

**Table 1:** Targeting posttranslational modifications of PD-1/PD-L1

Target	Posttranslational modifications	Regulator	Therapy	Preclinical tumor model	Ref. No
PD-L1 S176,T180,S184	Serine/Threonine phosphorylation	GSK3β	Gefitinib + anti-PD-1	TNBC	[27]
			Olaparib + anti–PD-1		
PD-L1 S195	Serine/Threonine phosphorylation	AMPK	Metformin + anti–CTLA4, D-mannose	BC, TNBC	[28, 155]
PD-L1 T210	Serine/Threonine phosphorylation	LRRK2	Vitamin B12	PDAC	[20]
PD-L1	Tyrosine phosphorylation	EGF	\	CSCC	[32]
PD-L1	Mono/Multiubiquitination	EGF	Gefitinib + SCH772984, PYR-41	CSCC	[33]
PD-L1	Poly-ubiquitination	β-TrCP	Resveratrol	TNBC	[27]
PD-L1	Poly-ubiquitination	Cullin3-SPOP	Palbociclib + anti–PD-1	BC, Colon cancer, CxCa	[35]
PD-L1	Poly- ubiquitination	STUB1	CMTM6 knockout, H1A	Melanoma	[45–47]
PD-L1	Ubiquitination	Casp8	\	Melanoma	[43, 44]
PD-L1	Ubiquitination	\	IFIT1	CRC	[36]
PD-L1	Poly- ubiquitination	SPOP	RIG-I knockout	CRC	[37]
PD-L1	Ubiquitination	RNF125	\	OSCC	[38]
PD-L1 K281	Poly- ubiquitination	HUWE1	PTPR + αCTLA-4	BC	[39]
PD-L1	Ubiquitination	VPRBP	USP2 knockout	Osteosarcoma	[40]
PD-L1	Ubiquitination	\	DHA	NSCLC, HCC	[41]
PD-L1	Poly- ubiquitination	Cullin3-SPOP	Canagliflozin	NSCLC	[48]
PD-L1	Ubiquitination	MIB2	\	Melanoma, CRC, NSCLC	[49]
PD-L1	Ubiquitination	\	PROTAC (21a, P22, AbTAC, CDTAC, ROTAC)	CRC, HCC, BC, NSCLC, bladder cancer, melanoma	[156–162]
PD-L1	Deubiquitination	CSN5	Curcumin + anti-CTLA4, PDIA6	TNBC, colon cancer, melanoma	[73–75]
PD-L1 K6, K11, K27, K29, K33	Deubiquitination	USP22	USP22 inhibitor: Rottlerin and Morusin	HCC, NSCLC	[76, 77]
PD-L1	Deubiquitination	USP9X	\	OSCC	[78]
PD-L1	Deubiquitination	UCHL1	\	NSCLC	[83]
PD-L1	Deubiquitination	OTUB1	\	BC	[84]
PD-L1 K270	Deubiquitination	USP2	USP2 knockout, USP2 inhibitor	CRC, PC	[79]
PD-L1	Deubiquitination	USP51	DHM	NSCLC	[80]
PD-L1	Deubiquitination	USP8	USP8 inhibitor + anti–PD-L1	PCA	[81]
PD-L1	Deubiquitination	USP7	A11 + PD-L1 mAb	BC, NSCLC, melanoma	[82]
PD-L1	Deubiquitination	USP20	\	BC	[85]
PD-L1	Deubiquitination	USP18	\	Bladder cancer	[86]
PD-L1 N192,N200	N-linked glycosylation	B3GNT3	STM108,Gefitinib	TNBC	[90]
PD-L1	N-linked glycosylation	Sigma1	IPAG	TNBC, PC	[91]
PD-L1	N-linked glycosylation	FKBP51 s	SAFit	GBM	[92]
PD-L1	N-linked glycosylation	GLT1D1	\	BCL	[93]
PD-L1	N-linked glycosylation	SLC35C1	\	LUAD	[94]
PD-L1	N-linked glycosylation	B4GALT1	\	LUAD	[95]
PD-L1	N-linked glycosylation	STT3	Etoposide + anti-Tim-3	BC, NPC, CxCa	[39, 96–99]
PD-L1	N-linked glycosylation	\	α-mangostin	TNBC	[188]
PD-L1	N-linked glycosylation	SEC61G	\	GBM	[100]
PD-L1	N-linked glycosylation	MCT4	\	TNBC	[101]
PD-L1	O-linked glycosylation	GALNT2/14	\	LUAD	[105]
PD-L1	O-linked glycosylation	GFAT1	\	Lung cancer	[106]
PD-L1	Ectodomain shedding	MMP9, 13	\	Mesenchymal stromal cells	[64]
PD-L1	Ectodomain shedding	MMP7, 13	CL82198, Paclitaxel	HNSCC	[65]
PD-L1	Ectodomain shedding	MMP2, 9, 13	HE4	OC	[66]
PD-L1	Disulfide bond formation	PDIA5	\	HCC, GBM, PC	[109]
PD-L1	Disulfide bond formation	ERO1-α	ERO1-α knockout	TNBC	[108]
PD-L1	S-palmitoylation	PPT1	PPT1 inhibitor	Melanoma, HCC	[190, 191]
PD-L1 C272	S-palmitoylation	ZDHHC9	2-BP	BC, Lung cancer	[112–115]
PD-L1 C272	S-palmitoylation	ZDHHC3	CPP-S1,2-BP, SP-PROTAC	CRC	[116, 196]
PD-L1	S-palmitoylation	FASN	DHA	Bladder cancer	[41, 117]
PD-L1	S-palmitoylation	DHHC5	DHA	PCA	[41]
PD-L1	ADP-Ribosylation	ARF6	\	PCA	[119, 120]
PD-L1	Acetylation	EGF	\	CSCC	[33]
PD-L1 K263	Acetylation	p300, HDAC2	HDAC2 inhibitor	TNBC	[125]
PD-L1	Acetylation	VPA	\	PCA	[126]
PD-L1	Autophagic degradation	ING4	CK2 inhibitor	NSCLC	[161]
PD-L1	SUMOylation	TRIM28	\	GC	[88]
PD-L1	ISGylation	ISG15	\	LUAD	[57]
PD-L1	UFMylation	UFL1, UFM1	UFSP2 inhibitor	TNBC, HCC	[58]
PD-L1 K162	Methylation	SETD7	\	NSCLC	[22]
PD-1 Y223,Y248	Tyrosine phosphorylation	Lck	Shp2	BL	[133]
PD-1 K48	Poly-ubiquitination	FBXO38	IL-2, CISH knockout	CRC, NSCLC	[13, 136]
PD-1	Ubiquitination	c-Cbl	\	CRC	[137]
PD-1 K48	Poly-ubiquitination	FBW7	Oridonin	NSCLC	[12]
PD-1	Ubiquitination	MDM2	\	Colon cancer	[138]
PD-1 N49,N74	N-linked glycosylation	Fut8	2 F-Fuc	Melanoma	[140]
PD-1	S-palmitoylation	DHHC9	\	CRC	[141]

**Table 2:** Clinical trials of targeting posttranslational modifications combined with PD-1/PD-L1 blockade

Therapy	Posttranslational modifications	Condition or disease	Efficacy & safety	Clinical trial No. & phase	Clinical trial status & cohort sizes	Ref. No
Avelumab 800 mg IV q2w + Talazoparib 1 mg po daily	Phosphorylation	Locally advanced (primary or recurrent) or metastatic solid tumors	ORR: NSCLC 16.7%, TNBC 18.2%, HR(+), ERBB2(-), and DDR(+) BC 34.8%, platinum-sensitive, BRCA wild-type OC 20.0%, UC 15%, DDR(+) mCRPC 11.1%; the most common ≥ grade 3 AEs: anemia 33.6%, thrombocytopenia 21.5%, and neutropenia 13.9%, 1 patient died due to acute respiratory syndrome.	NCT03330405, nonrandomized controlled phase I, II study	Terminated, 223 patients	[150]
Nivolumab+ TPST-1120	Phosphorylation	Advanced renal cell carcinoma (RCC), cholangiocarcinoma (CCA), or hepatocellular carcinoma	ORR: 23%, TPST-1120 ≥ 400 mg bid ORR: 38%; no grade 4/5 TRAEs or dose-limiting toxicities	NCT03829436, open-label, dose-escalation phase I study	Completed, 39 patients	[149]
Nivolumab IV administration every 14 days for 10 doses starting 14 days prior to IMRT + Cetuximab 7 doses + IMRT 5 fractions per week for 7 weeks for a dose of 70 Gy	Phosphorylation	Advanced HNSCC	The tested regimens was safe	NCT02764593, multiarm phase I trials	Completed, 10 patients	[147]
Durvalumab 1500 mg every 4 weeks for 8 cycles+ Cetuximab 400 mg/m^2^ 1 week before RT start followed by 250 mg/m^2^ weekly, for a maximum of 8 cycles	Phosphorylation	Locally advanced HNSCC	The median PFS, LRC and OS overlapped to 15 months, 1 and 2-year PFS rates: 77.7% and 58.3%; one case grade 4 mucositis lasting for 14 days	NCT03051906, single group, open-label, phase I, II study	Terminated, 9 patients	[146]
Nivolumab or Pembrolizumab + Metformin	Phosphorylation	Solid tumor malignancies	DCR: 22%, 12-month OS: low CAIX/I 53%, high CAIX/I 14.1%	NCT04114136, randomized, parallel assignment, open label, phase II study	Recruiting	[159]
Pembrolizumab 200 mg IV on day 1 of each 21-day cycle + Niraparib 200 mg daily po	Phosphorylation	Advanced or metastatic TNBC or recurrent ovarian carcinoma (OC)	ORR: OC patients 18%, 10 patients with TNBC 21%, BRCA mutation TNBC 47%; DCR: OC patients 65%, 23 patients with TNBC 49%, BRCA mutation TNBC 80%; PFS: OC patients 3.4 months, TNBC patients 2.3 months, BRCA mutation TNBC 8.3 months; 8% immune-related AE in OC patients, one TNBC patient death resulted from acute respiratory distress syndrome possibly related to treatment	NCT02657889, single group, open-label, phase I, II study	Completed, 9 patients with OC and 5 patients with TNBC in phase I, 53 patients with OC and 55 patients with TNBC in phase II	[151, 152]
Pembrolizumab2 mg/kg IV q3w + Erlotinib 150 mg daily po or Gefitinib 250 mg daily po	Phosphorylation	Unresectable or metastatic	Erlotinib: ORR: 41.7%, PFS: 19.5 months, OS: not reached (NR); Gefitinib: ORR: 14.3%, PFS: 1.4 months, OS: 13.0 months, grade 3/4 liver toxicity	NCT02039674, randomized, parallel assignment, open label, phase I, II study	Completed, 267 patients	[144]
Pembrolizumab 200 mg IV q3w + Cetuximab 400 mg/m^2^ IV 1 week followed by 250 mg/ m^2^ weekly, 3w	Phosphorylation	Recurrent/metastatic HNSCC	ORR: 45%, the most common grade 3,4 treatment-related AE was 9% oral mucositis, and serious.	NCT03082534, prospective, multicenter, open-label, nonrandomized, multiarm phase II trial	Active, not recruiting, 33 patients	[145]
Atezolizumab 1200 mg IV q3w + TPST-1120 1200 mg IV q3w+ Bevacizumab 15 mg/kg q3w	Phosphorylation	Advanced Liver Cancers	ORR:30%, β-catenin mutation ORR: 43%, PD-L1 negative ORR: 27%	NCT04524871, Phase Ib/II, open-label, multicenter, randomized umbrella study	Recruiting	[151]
Dostarlimab (TSR-042) 500 mg IV d1 + Niraparib 200 or 300 mg daily until PD or toxicity + Bevacizumab 15 mg/kg	Phosphorylation	Recurrent OC	ORR:17.9%, DCR:76.9%, PFS: 7.6 months; the most common grade ≥ 3 treatment-emergent adverse events (TEAEs) were hypertension (22.0%), fatigue (17.1%), and anemia (17.1%)	NCT03574779, randomized, parallel assignment, open label, phase I, II study	Recruiting, 41 patients	[157]
Durvalumab 1500 mg IV q4w + Olaparib 300 mg po bid	Phosphorylation	Recurrent endometrial cancer	ORR: 16%, PFS:3.4months, OS:8.0 months, grade 3 TRAEs: 16%	NCT03951415, single group assignment, open label, phase II study	Unknown status, 55 patients	[153]
Tislelizumab 2 mg/kg IV q3w + Pamiparib 20, 40, or 60 mg po bid	Phosphorylation	Advanced solid tumors	ORR: 20%, PFS: 92 days, OS: 388 days, grade ≥ 3 TRAEs: 12% anemia, 6% elevated elevated aspartate aminotransferase concentrations, 6% elevated γ-glutamyltransferase concentrations, 6% autoimmune hepatitis. No fatal AEs.	NCT02660034, nonrandomized, parallel assignment, open label, phase I study	Completed, 49 patients	[156]
Durvalumab 1.5 g IV q4w + Olaparib 300 mg po bid	Phosphorylation	Germline BRCA1-mutated or BRCA2-mutated(gBRCAm)metastatic BC, and OC	BC: PFS:8.2 months, OS:21.5 months, OC: gBRCAm expansion doublet: PFS: 15.0 months, OS: immature, ORR:92.2%; nongBRCAm doublet: PFS: 5.5 months, OS:26.1 months, ORR:34.4%; nongBRCAm triplet cohorts: PFS: 14.7 months, OS:31.9 months, ORR:87.1%	NCT02734004, nonrandomized, single group assignment, open label, phase I, II study	Active, not recruiting, BC:34 patients OC: 114 patients	[154, 155]
Part A (after progression on a previous EGFR TKI): Durvalumab 3 or 10 mg/kg q2w + Osimertinib 80 mg daily; Part B (first-line): Durvalumab 10 mg/kg q2w + Osimertinib 80 mg daily	Phosphorylation	EGFR-mutant lung cancer	Part A : ORR: 43%, DOR: 20.4 months; Part B : ORR: 82%, DOR: 7.1 months, PFS: 9.0 months; 35% interstitial lung disease overall	NCT02143466, multicenter, nonrandomized, parallel assignment, open label, phase I study	Part A: active, not recruiting; 23 patients, Part B: terminated, 11 patients	[189]
Avelumab (A) 10 mg/kg intravenously (IV) q2w + Palbociclib (P) 125 mg po daily (days 1–21 of each cycle) + Fulvestran (F) 500 mg intramuscularly (IM) once on days 1 and 15 in cycle 1 and 500 mg IM once on day 1 of each subsequent monthly cycle ; P + F; F	Ubiquitination	Hormone receptor-positive/HER2- Metastatic BC progressed on previous CDK4/6i and aromatase inhibitor therapy	PFS: A + P + F 8.1 months; P + F 4.6 months; F 4.8 months; ORR: A + P + F 13%; P + F 9%; F 7.3%; the most common grade 3/4 adverse event (AE) is neutropenia: A + P + F 49.1%, P + F 32.7%, F 0%	NCT03147287, randomized multicenter phase II PACE trial	Active, not recruiting, 220 patients	[169]
Avelumab 10 mg/kg IV q2w + Axitinib 3 mg po bid + Palbociclib 75 mg po daily (7 days off/21 days on)	Ubiquitination	Advanced NSCLC (no EGFR, ALK, or ROS1 alterations; PD-L1 unrestricted; ≤2 prior therapy lines)	The clinical benefit rate:53%; the most common grade 3/4 AE is neutropenia; one case grade 5 respiratory failure	NCT03386929, WIN Consortium multicenter phase I, II study	Terminated, 15 patients	[168]
Part A: SGN-2FF 1, 2, 5, 10, 15 g qd; 2 and 5 g b.i.d; Part B: SGN-2FF 5 g b.i.d; Part C: Pembrolizumab 200 mg IV + SGN-2FF 2, 5 g b.i.d	Glycosylation	Advanced solid tumors	Part A: 1/28 CR, 10/28 SD, Part B: 2/2 PD, Part C: 1/4 SD, 3/4 PD; grades 2–5 thromboembolic events 16%(5/32)	NCT02952989. open-label, multicenter, dose escalation, phase I study	Terminated, 46 patients Part A 33 patients, Part B 6 patients, Part C 7 patients	[178]
Atezolizumab 1200 mg IV q3w + Etoposide(EP) 100 mg/m^2^ on days 1–3 + Carboplatin under the AUC of 5 mg/mL/min	Glycosylation	Untreated extensive-stage SCLC	ORR: 71.6%, OS: 10.7 months, PFS: 5.5 months, serious AEs occurred in 29.9%	NCT04028050 multicenter, open-label, phase IIIb	Completed, 154 patients	[185]
Pembrolizumab 200 mg IV day1 + Etoposide 100 mg/m^2^ on days 1–3, q3w	Glycosylation	Extensive-stage SCLC	Pembrolizumab + EP: ORR 70.6%, twelve-month PFS estimates 13.6%;grade3/4 AEs: 76.7%, AEs led to death: 6.9% ; Placebo + EP: ORR 61.8%, twelve-month PFS estimates 3.1%, grade3/4 AEs: 74.9%, AEs led to death: 5.4%;	NCT03066778, randomized, double-blind, phase III KEYNOTE-604 study	Completed, 453 patients, pembrolizumab + EP 228 patients, placebo + EP 225 patients	[183]
Atezolizumab 1,200 mg IV d1 +Carboplatin 5 mg/mL/min + Etoposide 100 mg/m^2^ IV, days 1–3, q3w	Glycosylation	Untreated extensive-stage SCLC	Atezolizumab group : OS:12.3 months, PFS:5.2 months, DOR: 4.2 months; placebo group: OS:10.3 months, PFS:4.3 months, DOR: 3.9 months	NCT02763579, double-blind, placebo-controlled, phase III trial	Completed, atezolizumab group 201 patients, placebo group 202 patients	[186]
SCLC cohort: Tislelizumab 200 mg + Etoposide + Platinum q3w	Glycosylation	Advanced/metastatic lung cancer	SCLC cohort : ORR: 77%, PFS: 6.9 months, OS: 15.6 months,	NCT03432598, multicenter, open-label, phase II study	Completed, 54 patients	[188]
Serplulimab 4.5 mg/kg q3w + Carboplatin+ Etoposide	Glycosylation	Extensive-stage SCLC	ORR:80.2%, OS: 15.4 months, PFS: 5.7 months, TRAEs: grade ≥ 3 82.5%	NCT04063163, randomized, parallel assignment, double-blind, phase III study	Unknown status, 585 patients	[189]
Avelumab 800 mg IV q2w + Talazoparib 1 mg po daily	Phosphorylation	Advanced BC	\	NCT03964532, multi-institutional pilot phase I, II TALAVE trial	Active, not recruiting	\
Avelumab + Bempegaldesleukin (NKTR-214) + Talazoparib	Phosphorylation	Metastatic castration-resistant PC	\	NCT04052204, phase I, II study	Terminated	\
Nivolumab + Rucaparib	Phosphorylation	OC, ovarian epithelial cancer, fallopian tube cancer, adenocarcinoma of the appendix	\	NCT03824704, open label phase II, 2-stage, 2-cohort trial	Terminated	\
Nivolumab + Metformin	Phosphorylation	Stage III-IV NSCLC	\	NCT03048500, single group, open-label, phase II study	Unknown status	\
Nivolumab or Pembrolizumab + Recombinant human EGF-rP64K/montanide ISA 51 Vaccine	Phosphorylation	Advanced NSCLC or HNSCC	\	NCT02955290, nonrandomized, parallel assignment, open label, phase I, II study	Recruiting	\
Atezolizumab+ Olaparib	Phosphorylation	Locally advanced unresectable or metastatic non-HER2-positive BC	\	NCT02849496 randomized, crossover assignment, open label, phase II trial	Active, not recruiting	\
TSR-042 1000 mg IV d1, q6w + Niraparib 100 mg daily	Phosphorylation	First-line treatment of stage III or IV nonmucinous epithelial OC	\	NCT03602859, randomized, parallel assignment, triple (participant, care provider, investigator), phase III trial	Active, not recruiting	\
TSR-042 + Niraparib	Phosphorylation	Metastatic or recurrent endometrial or oOC	\	NCT03651206, randomized, parallel assignment, open label, phase II, III, study	Active, not recruiting	\
TSR-042 500 mg IV q3w (cycle 1-4) 1000 mg q6w (cycle 5 until PD or toxicity, up to 27 months) + Niraparib Weight ≥ 77 kg (kg) and platelet count ≥ 150,000/microliter (µL) at baseline: 300 mg daily; others: 200 mg daily	Phosphorylation	Platinum resistant OC	\	NCT03955471, single group assignment, open label, phase II, III, study	Terminated, 41 patients	\
Durvalumab + Olaparib	Phosphorylation	IDH mutated glioma	\	NCT03991832, nonrandomized, parallel assignment, open label, phase II, study	Recruiting	\
Sintilimab 1200 mg q3w + Metformin 1000 mg bid from day20	Phosphorylation	Extensive-stage SCLC	\	NCT03994744, open-label, single-arm, phase II study	Unknown status, 68 patients	\
PDR001 + Ribociclib	Ubiquitination	Metastatic HR + BC or metastatic OC	\	NCT03294694, nonrandomized, parallel assignment, open label, phase I study	Terminated, 33 patients	\
PDR001 + Ribociclib	Ubiquitination	NSCLC, HNSCC, ESCC, GC, CRC	\	NCT04000529, nonrandomized, parallel assignment, open label, phase I study	Terminated, 122 patients	\
SHR-1210 200 mg IV q2w + SHR6390 150 mg or 100 mg po daily with 3 weeks on and 1 week off	Ubiquitination	Advanced CRC, NSCLC or HCC	\	NCT03601598, single group assignment, open label, phase I, II study	Unknown status, 41 patients	\
Atezolizumab 1,200 mg IV d1 +Carboplatin 5 mg/mL/min + Etoposide 100 mg/m^2^ IV, days 1–3, q3w	Glycosylation	Untreated extensive-stage SCLC	\	NCT02748889, randomized, parallel assignment, open label, phase I, II study	Terminated	\
Nivolumab + RXC004	Palmitoylation	Advanced Malignancies	\	NCT03447470, nonrandomized, sequential assignment, open label, phase I study	Active, not recruiting	\
Nivolumab 480 mg q4w + RXC004 1.5 mg daily po	Palmitoylation	Advanced Colorectal Cancer	\	NCT04907539, open label, multicenter, multiarm, phase II study	Recruiting	\
Pembrolizumab + ETC-1,922,159	Palmitoylation	Advanced solid tumors	\	NCT02521844 nonrandomized, open-label, sequential evaluation of safety and dose, phase I study	Active, not recruiting	\
Pembrolizumab + CGX1321	Palmitoylation	Advanced GI tumors	\	NCT02675946 multicenter, open-label, dose escalation and expansion, phase I study	Unknown status	\

### PD-L1 phosphorylation-related therapeutic prospects and clinical transformation

#### Therapeutic promotion and clinical transformation of drugs that inhibit the EGFR pathway

Blocking the EGFR pathway is linked to an increase in PD-L1 levels in tumor cells, leading to improved outcomes from PD-1/PD-L1 blockade therapy in cancers such as breast cancer and NSCLC [27, 142]. Clinical trials are currently investigating combinations of EGFR inhibitors with PD-1/PD-L1 blockade therapies and EGF tumor vaccines (Table 2). However, the efficacy of these combinations is under scrutiny due to serious treatment-related toxicity, such as a 22% incidence of interstitial lung disease in the TATTON study [143] and a 71.4% rate of severe hepatotoxicity in another study involving pembrolizumab and gefitinib [144]. Despite these challenges, studies such as KEYNOTE-021 reported manageable toxicity and a 41.7% objective response rate (ORR) for the combination of pembrolizumab and erlotinib [144]. Additionally, cetuximab is being evaluated in trials for its potential to enhance immune checkpoint therapy in head and neck squamous cell carcinoma (HNSCC), as it has shown promising progression-free survival (PFS) rates and a 45% ORR [145, 146]. Final results on the safety and efficacy of these combination therapies are highly anticipated [147].

#### Therapeutic prospects and clinical transformation of PARP inhibitors

Jiao et al. reported that the drug olaparib, a PARP1 inhibitor, increases PD-L1 levels in cancer cells by deactivating GSK3β [148]. When olaparib was combined with anti-PD-L1 therapy, it was more effective in treating cancer in live models than when each drug was used alone [148]. As a result, many clinical trials are now testing combinations of PARP1 inhibitors and anti-PD-1/PD-L1 therapies (Table 2). Some of these trials have shown that these combinations are superior to standard treatments. For example, in a study involving patients with advanced kidney, bile duct, and liver cancers, one combination therapy resulted in a 23% ORR, which increased to 30% with a higher dose [149]. Common side effects included mild to moderate fatigue, diarrhea, and nausea [149]. Another trial showed that combining specific drugs for advanced liver cancer achieved a 30% response rate, which was better than the 13.3% response rate of the standard treatment [150]. This trial also revealed high response rates in patients with certain genetic markers, such as β-catenin, and even in those who did not express PD-L1, a target of the treatment [150]. Other trials exploring different combinations for breast and ovarian cancer have shown promising results with good tolerability [151–157].

#### Therapeutic promise and clinical transformation of metformin

Metformin activates AMPK, which phosphorylates PD-L1, disrupting its normal assembly and leading to its degradation. This interaction suggests that combining metformin with immune therapies such as CTLA4 blockers could improve cancer treatment outcomes [158]. However, metformin shows limited effectiveness against cancer under certain conditions where PD-L1 cannot be phosphorylated [158]. Current clinical trials are testing the effectiveness of combining metformin with anti-PD-1 therapy [159] (Table 2).

#### Therapeutic promise of LRRK2 inhibitors

LRRK2 is an enzyme that modifies PD-L1 by adding a phosphate group to it, which prevents PD-L1 from being broken down in cells. Inhibiting LRRK2 enhances the effects of PD-L1-targeted treatments in mice, increasing the therapeutic response. Adenosine cobalamin, a form of vitamin B12, effectively blocks LRRK2 and improves the response to PD-L1 immunotherapy in mice with pancreatic cancer. This approach, in which PD-L1 blockade is combined with LRRK2 inhibition, appears promising as a new treatment strategy for pancreatic cancer [20].

### PD-1/PD-L1 ubiquitination treatment prospects and clinical transformation

#### PROTACs targeting PD-1/PD-L1

The use of proteolysis-targeting chimeras (PROTACs), which target and degrade difficult-to-drug proteins, is a new method for cancer treatment [160, 161]. Wang et al. developed a PROTAC called 21a that breaks down the PD-L1 protein in various cancers [162]. Another PROTAC, P22, specifically disrupts the PD-1/PD-L1 interaction, enhancing therapeutic efficacy [163]. Cotton et al. proposed the use of antibody-based PROTACs (AbTACs), which use the E3 ligase RNF43 to target PD-L1 for lysosomal destruction [164]. Su et al. introduced carbon-based PROTACs (CDTACs), which also target PD-L1 but for proteasome degradation, showing promise in preclinical studies by inhibiting tumor growth and boosting the immune response [165]. Sun et al. developed ROTACs, a type of PROTAC that targets and degrades specific signaling molecules, using a chimera called R2PD1 to efficiently degrade PD-L1 in melanoma cells, outperforming existing treatments in activating immune responses and inhibiting tumor growth [166]. These developments suggest that PROTACs could significantly improve PD-1/PD-L1-targeted therapies for cancer [167].

#### Prospects and clinical translation of CDK4/CDK6 inhibitors

Research has revealed that CDK4/CDK6 inhibitors, by increasing CDH1 levels, promote the degradation of the SPOP protein, which in turn increases PD-L1 expression through a pathway involving cyclin D-CDK4. Using the CDK4/6 inhibitor palbociclib and PD-1 immunotherapy in a mouse colon cancer model resulted in significant tumor shrinkage and extended survival, highlighting a new regulatory mechanism involving cyclin kinase and ubiquitin ligase for PD-L1 [35]. In another discovery, Ding et al. reported that the diabetes drug canagliflozin disrupts the interaction between SGLT2 and PD-L1, allowing PD-L1 recognition and degradation by the Cullin 3-spopoe3 ligase and enhancing T-cell attack on tumor cells [48]. This finding illustrates a potential strategy for using existing drugs to decrease PD-L1 and boost immune responses against cancer. Additionally, Lin’s team identified PIK-93, a compound that increases the binding of PD-L1 to Cullin-4 A, thus improving the effectiveness of anti-PD-L1 immunotherapy [42]. These findings have propelled multiple clinical trials testing combinations of CDK4/6 inhibitors with PD-1/PD-L1 therapies [168, 169]. A phase I trial on advanced non-small cell lung cancer reported that 53% of patients showed clinical improvement and tolerated the treatment well, indicating a promising avenue for enhancing cancer immunotherapy [168] (Table 2).

#### Prospects and clinical translation of targeting the deubiquitination of PD-1/PD-L1

Zhang et al. discovered that the USP22 inhibitors Rottlerin and Morusin promote the breakdown of PD-L1 and Sirt1 proteins, suggesting a new method for cancer therapy [170]. In related research, combining a USP2 inhibitor with an anti-PD1 antibody led to complete tumor regression in models with functional p53, emphasizing the therapeutic potential of targeting protein stability [40]. Li et al. reported that the flavonoid dihydromyricetin (DHM) acts as a USP51 inhibitor, enhancing lung cancer cell sensitivity to chemotherapy by promoting PD-L1 degradation [80]. Similarly, a study on a USP8 inhibitor demonstrated its effectiveness in suppressing pancreatic tumor growth by activating killer T cells, especially when combined with anti-PD-L1 therapy [81]. Additionally, A11, an inhibitor of USP7, showed promising antitumor effects by blocking PD-L1’s ability to help tumors evade immune detection, and when combined with PD-1 antibody therapy, it showed enhanced antitumor activity [82]. Additionally, the application of the CSN5 inhibitor curcumin inhibited the ubiquitination of PD-L1, reduced PD-L1 expression, and increased the sensitivity of tumor cells to CTLA4 immunotherapy [73].

### Treatment prospects and clinical transformation associated with PD-1/PD-L1 glycosylation

#### PD-1 glycosylation enhances the binding of PD-1 to antibodies and reduces immune escape

The glycosylation of PD-1, particularly at the N58 site, significantly influences its interaction with certain antibodies. Glycosylation enhances the effectiveness of camrelizumab by improving its binding to PD-1 [171], whereas the interaction between cemiplimab and PD-1 mirrors that of camrelizumab [172–174]. Other antibodies, such as nivolumab and toripalimab, do not depend on glycosylation for their function [173, 175]. To address the challenges posed by the large size of typical IgG antibodies, researchers have developed smaller proteins, JYQ12 and JYQ12-2, from the extracellular domains of PD-1. These proteins, which are only 14–17 kDa and contain a single N-linked glycan chain, not only bind effectively to PD-L1 and PD-L2 but also enhance the proliferation of human T cells, showing promising potential for therapeutic and diagnostic applications in cancer immunotherapy [176].

#### Targeting the N-glycosylation site of PD-L1 blocks its interaction with PD-1

The glycosylation of PD-L1 strengthens its interaction with PD-1, suppressing immune responses and aiding tumor escape. To counter this, new drugs have been developed to target glycosylation sites. For example, the antibody STM108 targets glycosylated PD-L1 at specific sites (N35, N192, and N200), effectively blocking the PD-L1/PD-1 interaction. This finding demonstrates the potential of using glycosylation-specific antibodies in cancer therapy to prevent tumors from escaping the immune system [90].

#### Glycosylation of PD-L1 affects clinical immunohistochemistry

The glycosylation of PD-L1 can interfere with its detection by immunohistochemical antibodies, potentially causing false-negative results in tests that assess PD-L1 expression in cancer patients. This issue arises when glycosyl structures on the PD-L1 protein prevent antibody binding [90]. To address this issue, researchers have developed a method of removing these sugars—called deglycosylation—before testing. This technique significantly improves the accuracy of PD-L1 detection and correlates better with patients’ responses to anti-PD-1/PD-L1 therapies [177], and it has been patented (UTSC.P1325US. P1) due to its substantial clinical value.

#### Antitumor activity of the PD-L1 glycosylation inhibitor

Glycosylation inhibitors of PD-L1 are promising antitumor agents. In a phase I trial, the fucosylation inhibitor SGN-2FF combined with pembrolizumab yielded promising results in patients with advanced solid tumors, including a complete response in an HNSCC patient and significant tumor reduction in a TNBC patient (Table 2). However, the trial was stopped due to thromboembolism risks [178]. Newer inhibitors, such as A2F1P and B2FF1P, have shown greater effectiveness than SGN-2FF due to improved cellular retention and efficiency [179, 180]. Other developments include IPAG and SAFit, which inhibit PD-L1 glycosylation and degrade PD-L1 in cells, enhancing the potential for cancer therapy [84, 85]. Additionally, drugs such as BAY-876 inhibit glycolysis in TNBC, reducing PD-L1 glycosylation and enhancing the efficacy of anti-PD-L1 therapies [181]. These advancements demonstrate significant potential for developing drugs that target the PD-1/PD-L1 pathway, although safety and impact on normal tissues remain critical considerations [182].

Etoposide can inhibit the enzyme STT3, which is involved in N-glycosylation, through its anti-EMT effects, reducing PD-L1 levels and increasing the effectiveness of anti-Tim3 therapy [96]. Clinical trials of etoposide combined with anti-PD-1/PD-L1 immunotherapy are currently underway [183–189] (Table 2). In a phase III trial for extensive small cell lung cancer, compared with placebo, pembrolizumab combined with etoposide and platinum significantly improved 12-month PFS (13.6% vs. 3.1%, *P* = 0.0023), enhancing patient quality of life [183, 184]. Another study revealed that atezolizumab combined with carboplatin and etoposide increased overall survival (OS) to 12.3 months from 10.3 months with chemotherapy alone (*P* = 0.0154) and was well tolerated [185, 186]. Similarly, tislelizumab or serplulimab with the same regimen in different trials extended OS and PFS [188, 189].

#### Targeting the PD-L1 dimer inhibits PD-L1 function

PD-L1 can form homodimers and tetramers, and its complex glycosylation is linked to the homodimeric structure of its intracellular domain [190]. Natural compounds such as capsaicin, 6-gingerol, and curcumin may block the PD-1/PD-L1 interaction by targeting PD-L1 dimerization, enhancing anticancer immunity [191]. The small molecule BMS-202, with modified carbonyl to hydroxyl groups, produces two enantiomers, MS and MR, both of which disrupt PD-L1 function by targeting its dimerization [192]. Furthermore, compounds such as α-mangostin and ethanol extracts can inhibit PD-L1 glycosylation and promote its degradation by binding within the pocket of the PD-L1 dimer [193]. These findings from preclinical studies highlight the potential of designing inhibitors that target PD-L1 dimers to enhance immunotherapy efficacy.

### Treatment prospects and clinical transformation of PD-L1/PD-1 palmitoylation

Palmitoylation of PD-L1 stabilizes the protein, and targeting this modification enhances PD-L1 immunotherapy efficacy. Porcupine, a membrane-bound o-acyltransferase, is targeted by inhibitors such as LGK974, ETC-1,922,159, CGX1321, and RXC004 and is now in phase I trials [194]. These inhibitors have also been tested in combination with anti-PD-1/PD-L1 antibodies in clinical trials (Table 2). Research shows that chloroquine derivatives improve anti-PD-1 therapy in melanoma by targeting palmitoyl protein thioesterase 1 (PPT1) [195]. Combining PPT1 inhibitors with anti-PD-1 antibodies activates T cells, enhancing tumor immunity [196]. Innovative therapies include HHAT and APT1/2 inhibitors and 2-bromopalmitate (2-BP) in polymer-lipid hybrid nanoparticles (2-BP/CPT-PLNs) that replace anti-PD-L1 antibodies in immune checkpoint blockade, showing potent antitumor effects and improved survival in melanoma models [197–199]. Additionally, a novel peptide (CPP-S1) that inhibits PD-L1 palmitoylation and promotes its degradation offers another strategy to enhance immunotherapy efficacy [21].

Dai et al. demonstrated that targeting PD-L1 palmitoylation was more effective than direct targeting [200]. Additionally, Shi et al. created a PROTAC (SP-PROTAC) using an anastomotic peptide targeting the palmitoyl transferase ZDHHC3, which significantly reduced PD-L1 expression in a human cervical cancer cell line [201].

ZDHHC9 palmitoylates cGAS at Cys 404/405, enhancing its activation, while depalmitoylation byLYPLAL1 impairs cGAS function. TargetingLYPLAL1-mediated cGAS depalmitoylation could boost cGAS activation and improve antitumor immunotherapy efficacy [202]. As ZDHHC9 also affects PD-L1 palmitoylation, inhibitingLYPLAL1 might enhance overall immunotherapy outcomes.

### Therapeutic promise of other PD-L1/PD-1 posttranslational modifications

Several preclinical treatments targeting PD-1/PD-L1 posttranslational modifications are being developed. HDAC2 inhibitors combined with PD-1 antibodies have been shown to significantly delay tumor growth and improve survival in syngeneic MC38 mouse models [22]. JQ-1, which reduces PD-L1 expression through acetylation, shows potential for treating pancreatic cancer [127]. Pevonedistat, a NEDDylation inhibitor, is undergoing clinical trials for various cancers and may upregulate PD-L1 expression, although its effectiveness is still under study [203, 204]. Zhou et al. discovered a UFSP2 inhibitor that enhances UFMyation, decreases PD-L1 expression, and supports PD-1 blockade [59]. CK2 inhibitors trigger PD-L1 autophagic degradation and enhance antitumor immunotherapy when combined with PD-1 antibodies [61]. Additionally, paclitaxel, which increases MMP-13 in certain cancer cells, shows promise for head and neck cancer treatment when used with anti-PD-1 therapy [65]. These methods represent promising strategies for cancer immunotherapy.

## Summary and prospective

In this review, we summarize the PTMs of PD-1/PD-L1 and their regulatory mechanisms and propose new targets for biomarkers and combination therapies to enhance PD-1/PD-L1 blockade in immunotherapy. Despite these advances, many aspects of PD-1/PD-L1 PTMs remain elusive. For diagnosis, PD-L1 glycosylation can obscure antibody binding sites, causing false negatives [177]. Additionally, the degradation of the glycan region of the PD-L1 epitope may lead to a loss of staining on immunohistochemistry [205]. The absolute and effective glycosylation levels may also vary significantly [206]. In treatment contexts, PD-L1 PTMs can contribute to tumor progression. In addition to PD-1/PD-L1 blockade, PTMs are vital for antigen presentation, CAR-T-cell therapy, and vaccine development [207]. Innovations such as multifluorescence resonance energy transfer (multi-FRET) are enhancing PTM research, offering new avenues for advancing tumor immunotherapy [208].

## Data Availability

No datasets were generated or analysed during the current study.

## Abbreviations

2F‒Fuc: 2-fluoro-L-fucose
ADCC: Antibody-dependent cytotoxicity
ADPR: ADP-ribose
ARF9: ADP-ribosylation factor-6
BC: Breast cancer
BCL: B-cell lymphoma
BL: Burkitt lymphoma
CISH: Cytokine-inducible SH2 domain-containing protein
CPT: Camptothecin
CR: Complete response
CRC: Colorectal cancer
CSCC: Cutaneous squamous cell carcinoma
CTLs: Cytotoxic T lymphocytes
CxCa: Cervical cancer
Cys or C: Cysteine
DHHC: Aspartic acid-histidine-histidine-cysteine
DOR: Duration of response
ESCC: Esophageal squamous cell carcinoma
FASN: Fatty acid synthase
GBM: Glioblastoma
GC: Gastric cancer
Gly: Glycine
HCC: Hepatocellular carcinoma
HNSCC: Head and neck squamous cell carcinoma
HRD: Hyperprogressive disease
ICB: Immune checkpoint blockade
ICD: Immunogenic cell death
KATs: Lysine acetylases
LUAD: Lung adenocarcinoma
MD: Molecular dynamics
MHC: Major histocompatibility complex
MULTI-FRET: Multifluorescence resonance energy transfer
NPC: Nasopharyngeal carcinoma
NSCLC: Non-small cell lung cancer
NSQ: Nonsquamous
OC: Ovarian cancer
ORR: Objective response rate
OS: Overall survival
OSCC: Oral squamous cell carcinoma
PC: Prostate cancer
PCA: pancreatic cancer
PD-1: Programmed cell death protein 1
PD-L1: Programmed death-ligand 1
PDAC: Pancreatic ductal adenocarcinoma
PFS: Progression-free survival
PROTACs: PROteolysis-Targeting Chimeras
PTM: Posttranslational modification
SCLC: Small cell lung cancer
SD: Stable disease
Ser or S: Seronine
SQ: Squamous
TCR: T-cell antigen receptor
Thr or T: Threonine
TNBC: Triple-negative breast cancer
TRAE: Treatment-related adverse events
Tyr or Y: Tyrosine
ZAP70: ζchain-related tyrosine kinase 70
